# Living β-selective cyclopolymerization using Ru dithiolate catalysts†
†Electronic supplementary information (ESI) available: Experimental procedures, characterizations, ^1^H and ^13^C NMR spectra for new compounds and polymers, SEC traces, and other supporting experi-ments. See DOI: 10.1039/c9sc01326a


**DOI:** 10.1039/c9sc01326a

**Published:** 2019-07-22

**Authors:** Kijung Jung, Tonia S. Ahmed, Jaeho Lee, Jong-Chan Sung, Hyeyun Keum, Robert H. Grubbs, Tae-Lim Choi

**Affiliations:** a Department of Chemistry , Seoul National University , Seoul 08826 , Republic of Korea . Email: tlc@snu.ac.kr; b The Arnold and Mabel Beckman Laboratory of Chemical Synthesis , Division of Chemistry and Chemical Engineering , California Institute of Technology , Pasadena , California 91125 , USA

## Abstract

Cyclopolymerization (CP) of 1,6-heptadiyne derivatives is a powerful method for synthesizing conjugated polyenes containing five- or six-membered rings *via* α- or β-addition, respectively. Fifteen years of studies on CP have revealed that user-friendly Ru-based catalysts promoted only α-addition; however, we recently achieved β-selective regiocontrol to produce polyenes containing six-membered-rings, using a dithiolate-chelated Ru-based catalyst. Unfortunately, slow initiation and relatively low catalyst stability inevitably led to uncontrolled polymerization. Nevertheless, this investigation gave us some clues to how successful living polymerization could be achieved. Herein, we report living β-selective CP by rational engineering of the steric factor on monomer or catalyst structures. As a result, the molecular weight of the conjugated polymers from various monomers could be controlled with narrow dispersities, according to the catalyst loading. A mechanistic investigation by *in situ* kinetic studies using ^1^H NMR spectroscopy revealed that with appropriate pyridine additives, imposing a steric demand on either the monomer or the catalyst significantly improved the stability of the propagating carbene as well as the relative rates of initiation over propagation, thereby achieving living polymerization. Furthermore, we successfully prepared diblock and even triblock copolymers with a broad monomer scope.

## Abstract 2

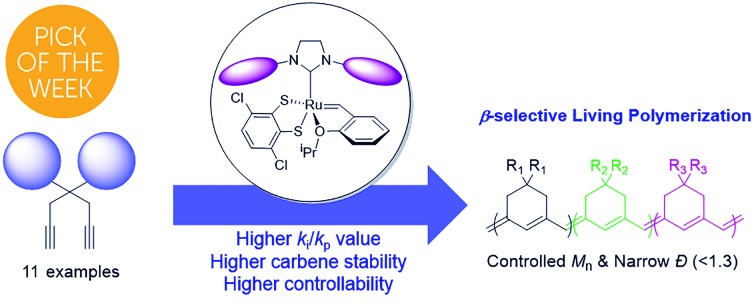
Herein, we demonstrate the first example of living β-selective CP by rational engineering of the steric factor on monomer or catalyst structures, along with a mechanistic investigation by *in situ* kinetic studies using ^1^H NMR spectroscopy.

## Introduction

Cyclopolymerization (CP) of terminal diynes *via* olefin metathesis is a powerful method for preparing conjugated polymers containing cycloalkene repeat units.^1^ After extensive early research on CP using ill-defined catalysts, such as Ziegler–Natta,^2^,^3^ MoCl_5_, and WCl_6_ catalysts,^4^–^8^ the first breakthrough on CP came with the development of well-defined catalysts by the Schrock group who reported the first living CP and the related comprehensive mechanistic details.^9^,^10^ The whole field expanded rapidly when Ru-based Grubbs^11^–^14^ and modified Grubbs catalysts (*e.g.*, Hoveyda–Grubbs^15^,^16^ and Buchmeiser catalysts^17^–^22^) showed excellent activity, selectivity, and stability for CP.

**Scheme 1:**
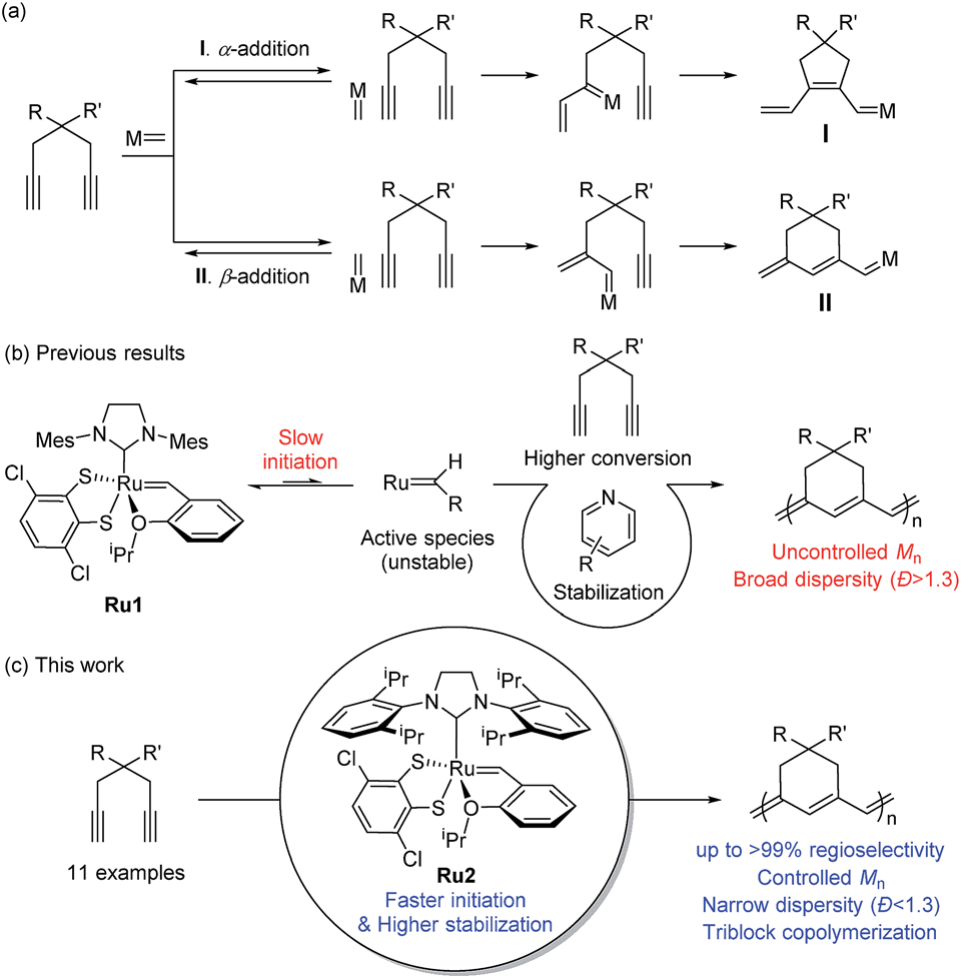
(a) Two possible pathways for CP of 1,6-heptadiynes, (b) proposed scheme showing the effects of the pyridine additive and the limitations of this method, and (c) living β-selective CP using fast-initiating **Ru2**.

To develop a more powerful CP, one should be able to control the reactivity to obtain high-molecular-weight (MW) polymers with a narrow distribution, as well as the regioselectivity to synthesize polymers with regular and predictable structures. As shown in Scheme 1a, there are two possible pathways in CP, α-addition and β-addition, resulting in a five- and six-membered ring repeat unit, respectively. The reaction pathway is determined by the orientation of the approaching metal alkylidenes to the terminal alkynes (Scheme 1a).^10^ Although the early-stage catalysts produced ill-defined regiorandom polyenes,^1^ the Buchmeiser group successfully demonstrated the first selective CP to generate five-membered rings *via* exclusive α-addition using Mo-based Schrock catalysts,^23^–^27^ and later, Ru-based Buchmeiser catalysts.^17^–^22^,^28^,^29^ Afterward, our group reported α-selective living CP to prepare various soluble polyacetylenes with complex architectures,^30^–^33^ and the Xie group demonstrated CP of functionalized monomers to generate polyacetylenes exerting high ionic conductivity,^34^–^36^ by using a user-friendly, fast-initiating third-generation Grubbs catalyst.^11^–^14^

The first β-selective CP producing six-membered rings was reported by the Schrock group in 1996, using modified Mo catalysts,^37^,^38^ but no follow-up studies have been reported. Recently, our group demonstrated the first β-selective CP using a user-friendly, Ru-based Grubbs *Z*-selective catalyst^39^ to generate conjugated polymers containing six-membered rings, with 67–95% β-selectivity.^40^ Notably, a new catalyst **Ru1** containing a dithiolate ligand, developed by the Hoveyda group,^41^ exerted far higher β-selectivity in CP of 1,6-heptadiyne monomers (85–99% β-selectivity), generating mainly six-membered conjugated polyenes bearing comparable thermal properties to the five-membered counterparts.^42^ However, these new polymers showed lower optical band-gaps than the five-membered analogues, thereby making them potentially more attractive materials in the electronic application.^42^ We also examined the origin of the exceptional regioselectivity using DFT calculations, concluding that **Ru1** which had adopted trigonal bipyramidal geometry would prefer β-addition due to electronic effects.^43^ However, both catalysts showed very slow initiation rate (*k*_i_) and relatively fast propagation rate (*k*_p_) leading to low *k*_i_/*k*_p_ values and poor MW control. Furthermore, the relatively low stability of the propagating carbene seemed to result in fast termination and broad dispersity (*Đ*) (Scheme 1b).^42^

β-Selective living CP is a much more challenging area, as there is only one example, using Schrock's Mo catalyst and just one monomer.^37^,^38^ Herein, we introduce two strategies to achieve β-selective living CP with user-friendly Ru catalysts: lowering *k*_p_ by introducing pyridine additives and sterically bulky substituents on monomers, and dramatically increasing *k*_i_ by employing a catalyst with a bulkier ligand. These strategies, combined with a synergetic effect of stabilized propagating species by steric demand, led to successful controlled polymerization with a broad monomer scope. Furthermore, we successfully demonstrated fully β-selective diblock and triblock copolymerizations (Scheme 1c). Finally, a mechanistic investigation using *in situ* kinetic experiments clarified the role of pyridine additives and allowed quantification of their effects by direct comparison of *k*_i_/*k*_p_ values.

## Results and discussion

**Fig. 1:**
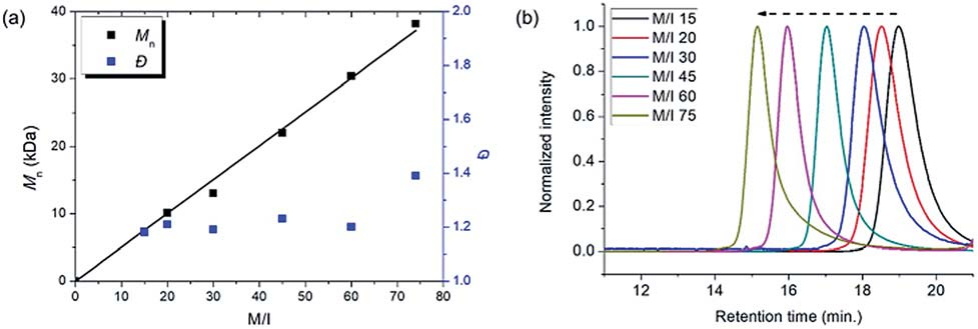
(a) Plots of the obtained *M*_n_*vs.* M/I (solid line shows a fit of the data) and the corresponding *Đ* values for **P2**, and (b) SEC traces of **P2** from entries 3–7 in Table 1.

To achieve living polymerization, high stability of the propagating species and a high *k*_i_/*k*_p_ value are crucial. In previous studies of ours and others, pyridine derivatives were found to coordinate to Grubbs catalysts,^11^,^44^ and in CP using **Ru1**, we discovered that pyridine additives coordinate to **Ru1** competitively with the monomer, thereby slowing down the polymerization and stabilizing the propagating species.^42^ However, living polymerization was not achieved, presumably due to low *k*_i_ and decomposition of **Ru1**. Since CP of monomer **M1** containing a *gem*-dimethyl group showed narrower dispersity (*Đ*) with the addition of 3,5-Cl_2_Py (reduced from 1.92 to 1.45),^42^ we expected that living polymerization might be achieved by introducing an even bulkier side chain, which would increase the stabilization on the propagating species and the *k*_i_/*k*_p_ value. Therefore, we synthesized **M2**, which replaced the ethyl ester and TMS side chains of **M1** with the sterically bulky *tert*-butyl ester and TIPS substituents (Table 1), and obtained conjugated polyene **P2** containing six-membered rings *via* exclusive β-addition. Without additives, CP of **M2** using **Ru1** at RT in DCM showed poor reactivity with less than 10% conversion (Table 1, entry 1). Although carrying out the reaction in THF at 70 °C increased the conversion to 94%, a broad *Đ* of 1.69 still implied uncontrolled CP (entry 2). Gratifyingly, with the addition of 3,5-Cl_2_Py, **P2** was synthesized at RT with higher conversion, and excellent molecular weight control was achieved, with a linear increase in *M*_n_ from 7.3 to 38.2 kDa with M/I between 15 and 75 (entries 3–7, Fig. 1a). Furthermore, the dispersities were less than 1.23, implying a successfully controlled polymerization, except for the highest-MW polymer (M/I of 75) which showed some tailing in size-exclusion chromatography (SEC) traces (entry 7, Fig. 1b).

**Table 1:** Living polymerization of **M2** using **Ru1** with 3,5-dichloropyridine

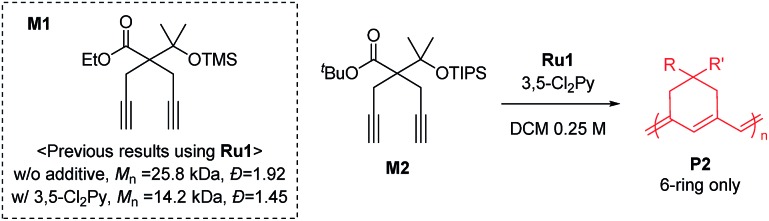							
Entry	M/I/add	Temp. (°C)	Time (h)	Conv.^*a*^ (%)	Yield^*b*^ (%)	*M*_n_ ^*c*^ (kDa)	*Đ* ^*c*^
1	30/1/—	25	1	<10	—	—	—
2^*d*^	30/1/—	70	3	94	69	18.2	1.69
3	15/1/10	25	1	>99	50	7.3	1.18
4	30/1/10	25	3	>99	81	13.0	1.19
5	45/1/15	25	3	>99	89	22.0	1.23
6	60/1/20	25	3	>99	78	30.4	1.20
7	75/1/25	20	8	98	79	38.2	1.39

^*a*^Determined by ^1^H NMR.

^*b*^Precipitated in MeOH at –78 °C.

^*c*^Determined by THF SEC calibrated using polystyrene standards.

^*d*^Conducted in THF.

**Scheme 2:**
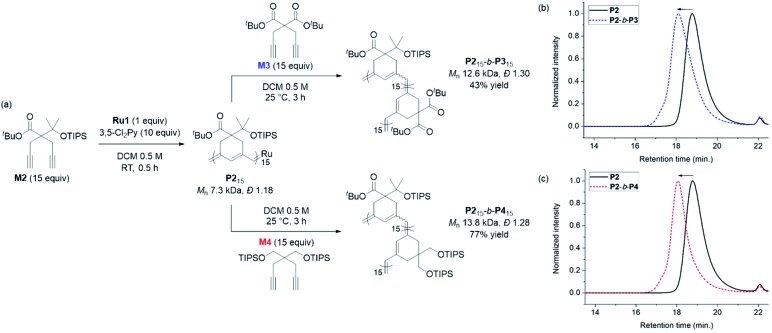
(a) Diblock copolymerization of with **M2** as the first monomer, and **M3** (above) and **M4** (below) as the second monomers. SEC traces of homopolymer **P2** and diblock copolymers: (b) **P2**-*b*-**P3**, and (c) **P2**-*b*-**P4**.

Having achieved β-selective living polymerization, we attempted diblock copolymerization at RT using **M2** as the first monomer (Scheme 2), because any of β-selective block copolymerization has never been reported. After complete consumption of 15 equiv. of **M2**, we added another 15 equiv. of **M3**, containing the di-*tert*-butyl malonate moiety, as the second monomer to prepare the fully conjugated polymer **P2**-*b*-**P3** by β-addition (Scheme 2a). Block copolymerization was confirmed by SEC analyses showing the complete shift of the traces from the **P2** homopolymer (7.3 kDa) to the block copolymer (12.6 kDa), with a narrow dispersity (1.30, Scheme 2b).

An analogous diblock copolymerization was successful as well when 15 equiv. of **M4**, containing bis-silylether, was introduced as the second monomer, to afford **P2**-*b*-**P4**, with a *M*_n_ of 13.8 kDa and *Đ* of 1.28, which was verified by SEC analyses (Scheme 2c). Remarkably, these two diblock copolymers showed perfect β-selectivity, confirmed by ^13^C NMR measurements. Although living homopolymerizations of **M3** and **M4** using **Ru1** were not possible in our previous work,^42^ we were able to prepare well-defined diblock copolymers from the **P2** macroinitiator as the initiation and stability of the living chain end were established in the first block.

To understand the origin of the successful living polymerization, we conducted a mechanistic investigation using *in situ* NMR analysis by monitoring initiation and propagation of **Ru1** during CP of **M2** (M/I = 20 in 0.1 M DCM-*d2*). With the pyridine additives, the signal intensity of the new propagating carbene proton gradually increased by up to 74% during the first 40 minutes, whereas that from a smaller monomer, diethyl malonate-derived **M5**, increased by only 56% in 5 minutes and then decreased continuously to 32% at 25 minutes (see ESI† for details).‡
‡Without pyridine additives, the propagating carbenes from **Ru1** were not observable, so it was impossible to compare the stability of the propagating carbene with and without additives. This result supports our hypothesis that a bulkier monomer enhances the stability of the propagating species. Furthermore, from this *in situ* NMR monitoring, *k*_i_ and *k*_p_ for CP of **M2** were obtained with and without the 3-ClPy additive (Fig. S3a and b†). With 7 equiv. of 3-ClPy, *k*_p_ was approximately three times lower than without the pyridine additive (0.15 *vs.* 0.05 min^–1^), due to competitive coordination to form a dormant 18e^–^ species, while *k*_i_ did not significantly change. Therefore, the overall *k*_i_/*k*_p_ value increased by 2.3 times with the use of 3-ClPy (3.07 *vs.* 1.31). SEC analyses of the resulting **P2**s demonstrated that an *M*_n_ of 10.1 kDa (close to the theoretical value, 8.1 kDa) with a narrow *Đ* (1.21) was obtained using 3-ClPy, whereas an unusually high *M*_n_ of 38.0 kDa and a broad *Đ* (2.05) were found without addition of 3-ClPy (Fig. S3c†). In short, sterically bulky monomers and pyridine additives increase the stability of the propagating species (from **Ru1**) as well as the *k*_i_/*k*_p_ value, thereby promoting living polymerization.

**Fig. 2:**
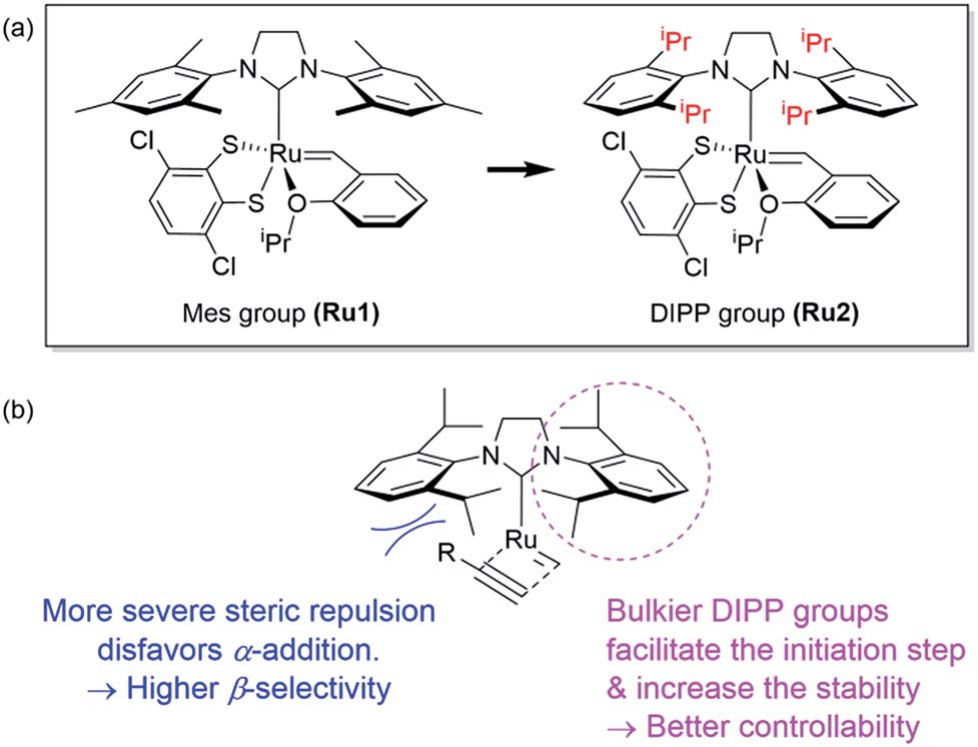
(a) Modifying ligands for living polymerization, and (b) model for improved β-selectivity and controllability of CP using **Ru2**.

However, living polymerization of monomers with smaller substituents was not possible using this approach.^42^ We then envisioned that a faster initiating β-selective catalyst would be necessary to increase the scope of suitable monomers for living polymerization. The Wagener group improved the initiation efficiencies of Grubbs and Hoveyda-Grubbs catalysts by replacing mesityl groups in the *N*-heterocyclic carbene (NHC) ligand with much bulkier *N*-2,6-diisopropylphenyl (DIPP) groups, facilitating dissociation of the Ru–O bond.^45^ This inspired us to use new dithiolate catalyst **Ru2**^46^ containing the bulky DIPP NHC ligand (Fig. 2a) to promote living CP with an even higher β-selectivity with a broader monomer scope, as a result of steric repulsion between the DIPP group and monomer substituents in the metallacyclobutene intermediates (Fig. 2b).

**Fig. 3:**
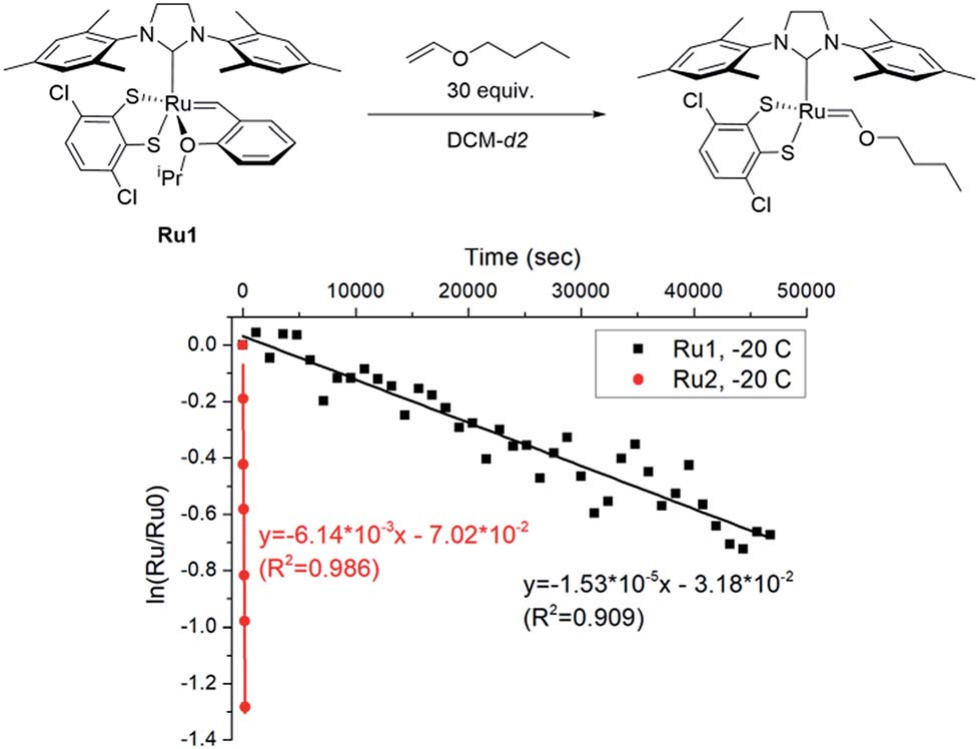
The plot of ln([Ru]/[Ru]_0_) *vs.* time to measure the initiation rates of **Ru1-2**^46^ at –20 °C by monitoring the disappearance of the benzylidene signal using ^1^H NMR.

To explore the effect of the DIPP group on the initiation, we measured the initiation rate of **Ru1** (*k*_i,_**_Ru1_**) to compare it with the recently reported value of **Ru2** (*k*_i,_**_Ru2_**).^46^ Following the reported protocol, the consumption of **Ru1** was monitored by ^1^H NMR spectroscopy upon addition of butyl vinyl ether at –20 °C, and *k*_i,_**_Ru1_** was determined to be 1.53 × 10^–5^ s^–1^, which is about 400 times slower than *k*_i,_**_Ru2_** measured under identical conditions (Fig. 3). Therefore, much faster-initiating **Ru2** should be an effective catalyst for living β-selective CP.

To test β-selective CP using **Ru2**, we chose diethyl dipropargyl malonate (**M5**) as a model monomer because its β-selectivity can be easily measured using ^1^H NMR (Table 2). The reaction with an M/I of 30, without an additive, was completed in just one minute with a high β-selectivity of 95% (Table 2, entry 1). This indicated that **Ru2** was more active and highly β-selective compared with **Ru1**, which, when used under the same reaction conditions, resulted in a 76% conversion and 85% β-selectivity after 1 hour.^42^ However, the propagation of CP using **Ru2**, compared to the initiation, was too fast for controlled polymerization; thus, *M*_n_ and *Đ* values were higher than expected. To lower the *k*_p_, 6 equiv. (with respect to the catalyst), of 3,5-Cl_2_Py, the optimal additive for **Ru1**, was used, but *Đ* was still broad (1.41), implying that 3,5-Cl_2_Py was not effective for **Ru2** (entry 2).

**Table 2:** Optimization of pyridine additives for CP of **M5** using **Ru2**

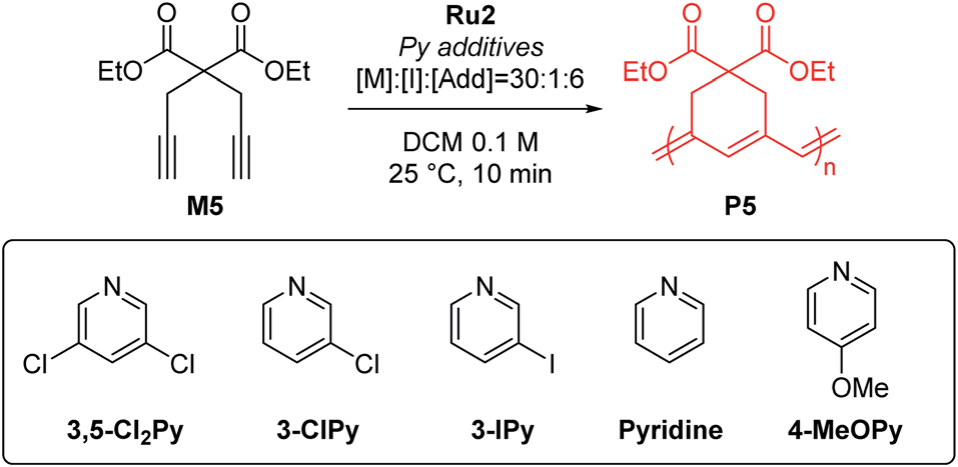						
Entry	Additive	Conv.^*a*^ (%)	Yield^*b*^ (%)	*M*_n_ ^*c*^ (kDa)	*Đ* ^*c*^	β-selectivity^*a*^ (%)
1	—	>99	99	13.6	1.86	95
2	3,5-Cl_2_Py	>99	99	14.2	1.41	95
3	3-ClPy	>99	92	10.8	1.19	96
4	3-IPy	>99	72	9.6	1.19	94
5	Pyridine	>99	77	9.6	1.11	97
6	4-MeOPy	>99	78	9.1	1.10	95

^*a*^Determined by ^1^H NMR.

^*b*^Precipitated in hexane at –78 °C.

^*c*^Determined by THF SEC, calibrated using polystyrene standards.

To our delight, the use of sterically less bulky and more strongly-binding mono-halogenated pyridine derivatives such as 3-ClPy and 3-IPy led to much narrower dispersities (*Đ* of 1.19, entries 3 and 4). These results led us to speculate that the binding affinity of the additives affected the controllability of CP, so we tried more basic ligands such as pyridine and 4-MeOPy. As a result, we observed controlled polymerizations with even narrower dispersities (*Đ* of 1.11 and 1.10, respectively, entries 5 and 6), and the highest β-selectivity, 97% in the pyridine case.

**Fig. 4:**
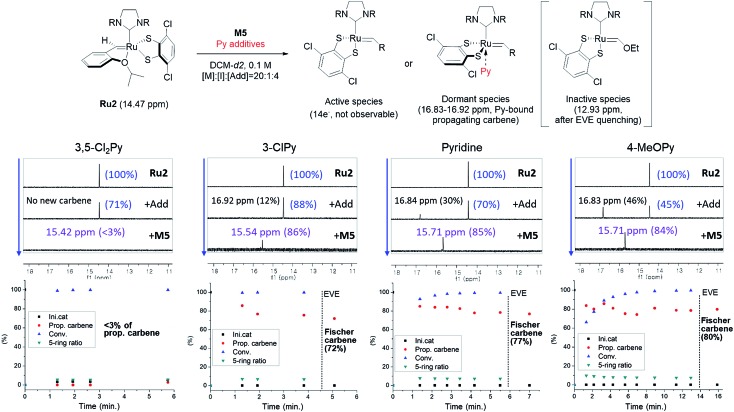
Scheme for the ^1^H NMR kinetic experiments (top), monitoring the changes in carbene proton signals during CP of **M5** using **Ru2** (middle) and their corresponding plots in real time (bottom).

**Fig. 5:**
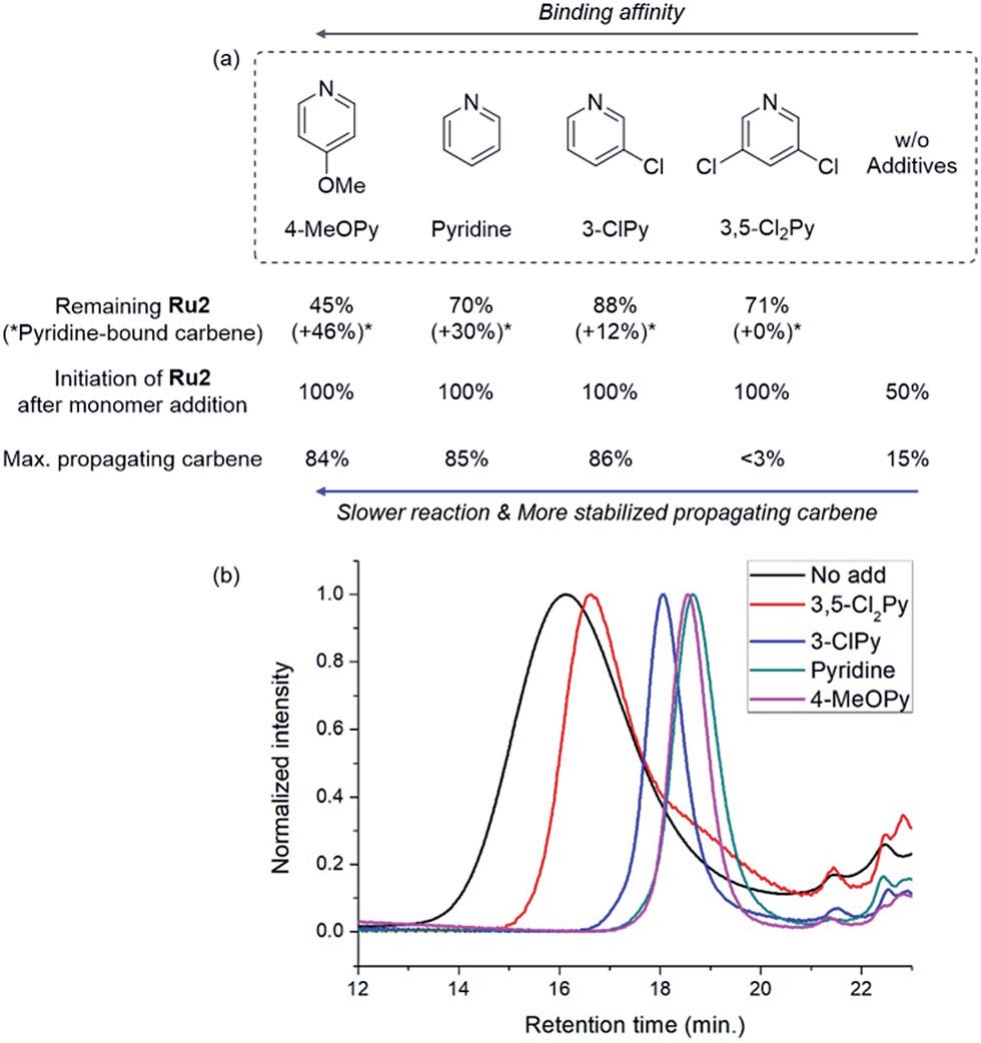
(a) Summary of the results from the kinetic experiments showing the relationship with the binding affinity of the additives and (b) THF-SEC traces of the corresponding polymers.

To investigate how various pyridine additives with different electronic properties affected the efficiency and selectivity of CP, we conducted *in situ* kinetic studies using ^1^H NMR, and monitored changes in the propagating carbene protons with four pyridine additives (M/I = 20 in 0.1 M DCM-*d2*, Fig. 4). First, upon addition of 3,5-Cl_2_Py to the **Ru2**, the carbene proton signal for **Ru2** at 14.47 ppm decreased to 71% without generating a new carbene proton signal. After the addition of the monomer **M5**, **Ru2** fully initiated with complete conversion of **M5** in 80 seconds. This indicates superior reactivity of **Ru2** to CP since for **Ru1**, only half of the catalyst initiated after 80 seconds, and it took 10 minutes for the complete conversion of **M5** (see ESI† for details). However, virtually no propagating carbene proton or Fischer carbene proton signal was detected during polymerization or after quenching with ether vinyl ether (EVE), suggesting complete decomposition of the catalyst. Therefore, we concluded that coordination of the bulkier and less basic 3,5-Cl_2_Py to **Ru2** was inefficient, resulting in a broad dispersity due to the failure of stabilizing the active species (*Đ* of 1.86, Fig. 5b).

**Fig. 6:**
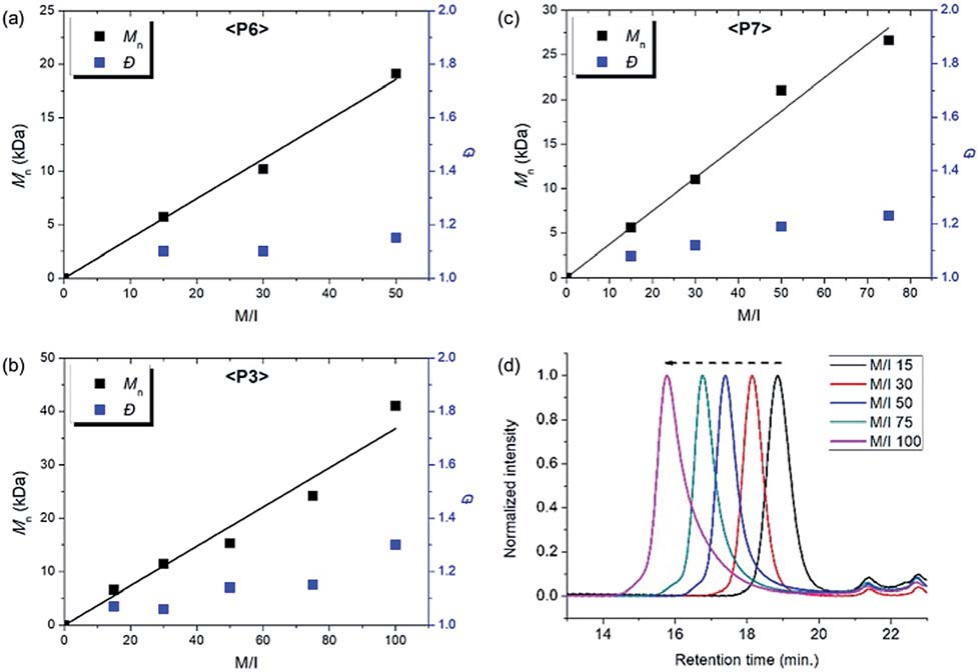
Plots of *M*_n_*vs.* M/I and corresponding *Đ* values of (a) **P6**, (b) **P3**, (c) **P7**, and (d) SEC traces of **P3** from entries 7–11.

When the stronger ligand 3-ClPy was added, the amount of **Ru2** dropped to 88% while at the same time, a new carbene proton signal equivalent to 12% of the initial **Ru2** signal appeared at 16.92 ppm. This new carbene is thought to be pyridine-bound **Ru2**, given the downfield shift, and the sum of two carbene proton signals was 100%, suggesting no decomposition of the catalyst. Upon the addition of **M5**, the signals of **M5** and both carbene protons disappeared in 90 seconds, and a new propagating carbene proton signal appeared at 15.54 ppm, with 86% intensity relative to the initial **Ru2** signal. When we added the more strongly binding pyridine and 4-methoxypyridine to **Ru2**, new carbene proton signals at 16.8 ppm were observed with higher relative intensities of 30% and 46%, respectively. These two effective ligands slowed down the propagation and stabilized the resulting propagating carbenes (15.71 ppm), with 85% and 84% intensities, respectively, which persisted throughout the reactions. Given that the propagating carbene proton signal in the reaction with **Ru1** and **M5** reached only 56% of the initial **Ru1** signal intensity, the higher values (up to 86%) observed with **Ru2** strongly support that the bulky DIPP ligand efficiently stabilizes the propagating species, thereby improving the controllability, with *Đ* as low as 1.11. A summary of the kinetic experiments is shown in Fig. 6a, demonstrating that the stronger electron-donating pyridine additives tend to stabilize the propagating carbenes more efficiently by forming 16 or 18e^–^ dormant states. This is well-reflected in the corresponding SEC traces showing a narrow Gaussian distribution for pyridine and 4-MeOPy, but broad dispersities with long tailings due to chain termination for the cases with no additive or weakly coordinating 3,5-Cl_2_Py (Fig. 5b). We selected pyridine as the optimal additive because it gave the highest β-selectivity as well as a low *Đ* (Table 2).

Using pyridine as an additive, we investigated controlled β-selective CP of various malonate-type monomers (Table 3). We observed the complete conversion of **M5**, with controlled *M*_n_ and narrow dispersity (1.15) for M/Is of up to 50, and ^13^C NMR analysis confirmed the high β-selectivity (94%), showing the six-membered ring on the polymer backbone (entry 1). For the higher M/I of 75, we obtained **P5** with an expected *M*_n_ of 21 kDa, but a broadening appeared in the SEC trace with *Đ* of 1.50, even after several optimizations (entry 2). Additionally, a higher-molecular-weight shoulder corresponding to doubling of the molecular weight appeared in the SEC trace of the resulting polymer, presumably due to the bimolecular decomposition of **Ru2** after prolonged reaction time.^47^ We were able to solve this problem by reducing the reaction time or concentration (see ESI† for details). Using monomer **M6**, containing an isopropyl group, CP proceeded with high reactivity for M/Is of 15 to 75 and a corresponding linear increase in *M*_n_ from 5.7 to 24.4 kDa, while retaining high β-selectivity (94%) and narrow *Đ* values (<1.15), except for the highest DP polymer (M/I of 75) where a severe broadening in the SEC trace was observed (entries 3–6, Fig. 6a). Taking a lesson from our previous work that introducing a sterically bulkier substituent improved both the stability of the propagating species and β-selectivity, we chose monomer **M3** with a much bulkier *tert*-butyl group, which was polymerized to yield **P3** with a higher β-selectivity of 97%. More importantly, we observed improved polymerization efficiency and controllability, generating **P3** with a linear increase in *M*_n_ up to 41 kDa and narrow dispersities (*Đ* of 1.06–1.30 for M/Is 15–100, entries 7–11, Fig. 6b and d). Maximizing the steric bulkiness by introducing an adamantyl group in **M7**, we successfully conducted CP with complete conversion, producing **P7** with a linear increase in *M*_n_ (6–27 kDa) and narrow *Đ* (1.12–1.23) for M/Is of 15 to 75 (entries 12–15, Fig. 6c). Notably, **P7** contained only six-membered ring repeat units *via* exclusive β-addition, as determined by ^13^C NMR analysis. To broaden the monomer scope, we tested CP using **Ru2** and monomers containing various non-malonate functional groups for M/Is of 30 and 50 (Table 4). CP of **M8**, containing the small ethyl ether group, showed high reactivity with complete conversion, and a high β-selectivity of 93%, but uncontrolled *M*_n_ with relatively broad dispersities (entries 1 and 2). Bulky **M9** and **M4**, containing silylether groups, showed not only excellent reactivity and β-selectivity (99%) but also great controllability with narrow *Đ*s, except for a slight broadening in the CP of **M4**, with a M/I of 50 (entries 3–6). **M1** was another successful example with an excellent conversion for both M/Is of 30 and 50, producing **P1** with a controlled *M*_n_ and narrow dispersity (<1.20), along with an excellent β-selectivity of 99% (entries 7 and 8). Furthermore, **M10**, with a pivaloyl group, reacted efficiently with excellent conversion, controlled *M*_n_, and narrow dispersity (<1.20) for both M/Is of 30 and 50, despite showing a moderate β-selectivity of 74% (entries 9 and 10). Lastly, amide group-containing **M11** showed complete conversion for an M/I of 30 with a narrow *Đ* and a good β-selectivity of 84%, but the reactivity decreased at higher M/I, resulting in only 74% conversion and uncontrolled *M*_n_ and *Đ* (entries 11 and 12).

**Table 3:** CP of various malonate type 1,6-heptadiyne monomers using **Ru2** and pyridine

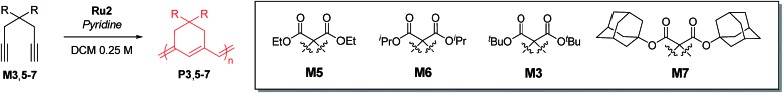										
Entry	Monomer	M/I/add	Temp. (°C)	Time	Conv.^*a*^ (%)	Yield^*b*^ (%)	*M*_n,theo_ (kDa)	*M*_n_ ^*c*^ (kDa)	*Đ* ^*c*^	β-selectivity^*d*^ (%)
1	**M5**	50/1/10	25	15 min	>99	86	11.8	15.5	1.15	94
2		75/1/10	20	30 min	92	87	16.3	20.7	1.50	
3	**M6**	15/1/5	25	3 min	>99	86	4.0	5.7	1.10	
4		30/1/10	25	10 min	>99	64	7.9	10.2	1.10	
5		50/1/10	20	30 min	>99	85	13.2	19.1	1.15	94
6		75/1/10	15	4 h	85	75	16.9	24.4	1.94	
7	**M3**	15/1/5	25	10 min	>99	56	4.4	6.6	1.07	
8		30/1/10	25	30 min	>99	70	8.8	11.4	1.06	
9		50/1/15	25	2 h	>99	81	14.6	15.3	1.14	97
10		75/1/20	25	3 h	>99	82	21.9	24.2	1.15	
11		100/1/25	25	4 h	>99	97	29.2	41.0	1.30	
12	**M7**	15/1/5	25	10 min	>99	62	6.7	5.6	1.08	
13		30/1/10	25	20 min	>99	72	13.5	11.0	1.12	
14		50/1/15	25	2 h	>99	86	22.4	21.0	1.19	>99
15		75/1/10	15	4 h	>99	76	33.7	26.6	1.23	

^*a*^Determined by ^1^H NMR.

^*b*^Precipitated in hexane at –78 °C.

^*c*^Determined by THF SEC calibrated using polystyrene standards.

^*d*^Determined by ^13^C NMR.

**Table 4:** CP of various 1,6-heptadiynes using **Ru2** and pyridine

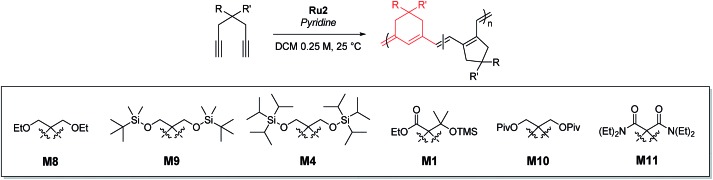									
Entry	Monomer	M/I/add	Time (h)	Conv.^*a*^ (%)	Yield^*b*^ (%)	*M*_n,theo_ (kDa)	*M*_n_ ^*c*^ (kDa)	*Đ* ^*c*^	β-selectivity^*d*^ (%)
1	**M8**	30/1/10	0.5	>99	83	6.3	8.1	1.36	93
2		50/1/15	1	>99	85	10.4	9.4	1.65	
3	**M9**	30/1/10	1	>99	82	11.4	15.4	1.11	>99
4		50/1/15	2	>99	73	19.0	26.5	1.17	
5	**M4**	30/1/10	2	>99	66	14.0	16.3	1.11	>99
6		50/1/15	3	97	60	22.6	26.2	1.35	
7	**M1**	30/1/10	1	>99	61	8.8	7.8	1.20	>99
8		50/1/15	3	>99	77	14.7	14.2	1.18	
9	**M10**	30/1/10	2	>99	75	9.6	11.3	1.11	74
10		50/1/15	4	>99	78	16.0	21.7	1.18	
11^*e*^	**M11**	30/1/10	1.5	>99	99	8.7	8.4	1.18	84
12^*e*^ ^,^^*f*^		50/1/15	2	74	65	10.8	7.7	1.37	

^*a*^Determined by ^1^H NMR.

^*b*^Precipitated in MeOH at –78 °C.

^*c*^Determined by THF SEC calibrated using polystyrene standards.

^*d*^Determined by ^13^C NMR.

^*e*^Precipitated in hexane at –78 °C.

^*f*^Conducted at 15 °C.

**Scheme 3:**
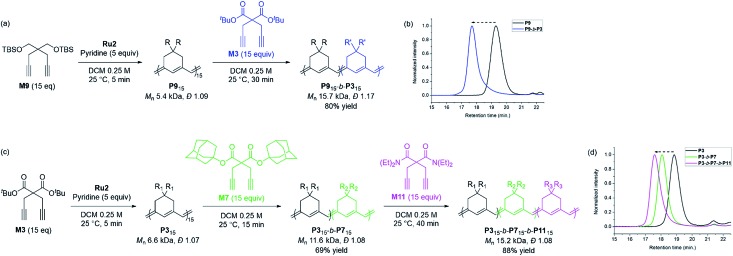
Diblock and triblock copolymerization for exclusively β-selective conjugated polyenes (a and c), and THF-SEC traces of the corresponding polymers (b and d).

Based on the successfully controlled polymerization, we tried another block copolymerization to determine if living polymerization was feasible with **Ru2**. Initially, **M9** was used as the first monomer to form **P9** with an *M*_n_ of 5.4 kDa and *Đ* of 1.09, after which 15 equiv. of **M3** was added to successfully produce **P9**-*b*-**P3** (*M*_n_ 15.7 kDa, *Đ* 1.17), detected by SEC analysis with a complete shift of the traces (Scheme 3a and b). Using the same procedure, we synthesized another diblock copolymer using **M3** as the first monomer, followed by the addition of 15 equiv. of **M7** as the second monomer, to form **P3**-*b*-**P7** with an *M*_n_ of 11.6 kDa and a narrow dispersity of 1.08 (Scheme 3c). To this living polymer end, we further added 15 equiv. of a third monomer, **M11**, which resulted in complete conversion to produce **P3**-*b*-**P7**-*b*-**P11**, showing a complete shift of SEC trace, with *M*_n_ of 15.2 kDa and *Đ* of 1.08 in a good yield of 88% (Scheme 3d). This remarkable success with diblock and triblock copolymerizations with a broad monomer scope and narrow *Đ* suggests the superior versatility of **Ru2** in β-selective living/controlled polymerization, compared with **Ru1**, which had a narrower scope.

## Conclusions

In conclusion, we successfully performed β-selective living/controlled CP using two Ru dithiolate catalysts to prepare various conjugated polyenes, bearing mostly six-membered ring repeat units. The high controllability was achieved by maximizing the steric demands on either the monomer or the catalyst, which improved the stability of the propagating species with the aid of pyridine additives. **Ru1** containing less bulky NHC ligands required the extremely bulky monomer **M2** for controlled polymerization. On the other hand, **Ru2**, already containing a bulky ligand, demonstrated much faster initiation and intrinsically greater stabilization of the propagating species, whereby a versatile living polymerization with a broader monomer scope was possible. Furthermore, we systematically studied the effect of pyridine additives and changing the catalyst by *in situ*^1^H NMR kinetic experiments. Particularly, we found that ligands which coordinate more strongly to **Ru2** better stabilized the propagating species and promoted better living/controlled CP. More significantly, we achieved several β-selective diblock and triblock copolymerizations, for the first time. In short, we achieved a rare β-selective living CP by analyzing the mechanistic details and kinetic parameters, and we expect this study to increase the insight into and versatility of Ru-catalyzed polymerizations.

## Conflicts of interest

There are no conflicts to declare.

## Supplementary Material

Supplementary information
