# Supplementary material for: Living β-selective cyclopolymerization using Ru dithiolate catalysts
Source: Chem Sci. 2019 Jul 22;10(39):8955–63. doi: 10.1039/c9sc01326a (PMC6855257; doi:10.1039/c9sc01326a)
Supplement: Supplementary file 1 [file SC-010-C9SC01326A-s001.pdf]

Electronic Supplementary Material (ESI) for Chemical Science.
This journal is © The Royal Society of Chemistry 2019

Supporting Information

Living *β*-Selective Cyclopolymerization Using Ru Dithiolate Catalysts

Kijung Jung,[†] Tonia S. Ahmed,[‡] Jaeho Lee,[†] Jong-Chan Sung,[†] Hyeyun Keum,[†] Robert H. Grubbs,[‡] and Tae-Lim Choi*,[†]

[†]Department of Chemistry, Seoul National University, Seoul 08826, Republic of Korea

[‡]The Arnold and Mabel Beckman Laboratory of Chemical Synthesis, Division of Chemistry and Chemical Engineering. California Institute of Technology, Pasadena, California 91125, United States

E-mail: tlc@snu.ac.kr

Table of Contents

1. General experimental....S3

2. Experimental procedures for the preparation of the monomers....S4–S5

3. General procedure for the cyclopolymerization....S5

4. Calculation of the regioselectivity for **P5** using ^{1}H and ^{13}C NMR....S6

5. *In situ* NMR experiment: procedure and data....S7–S8

6. SEC traces of the polymers....S9–S12

7. ^{1}H and ^{13}C NMR characterization of the polymers....S13

8. ^{1}H and ^{13}C NMR spectra of the polymers....S14–S29

9. ^{1}H and ^{13}C NMR spectra of the monomers....S30–S33

10. MALDI-TOF spectrum of **P5** (end-group fidelity)....S34

11. References....S35

1. General experimental

Materials

All reagents which are commercially available from Sigma-Aldrich®, Tokyo Chemical Industry Co. Ltd., Acros Organics, Alfa Aesar®, without additional notes, were used without further purification. Dichloromethane for the polymerization were purified by Glass Contour Organic Solvent Purification System, and degassed further by Ar bubbling for 10 minutes before performing reactions. Thin-layer chromatography (TLC) was carried out on MERCK TLC silica gel 60 F254 and flash column chromatography was performed using MERCK silica gel 60 (0.040~0.063 mm).

Characterization

^{1}H-NMR and ^{13}C-NMR were recorded by Varian/Oxford As-500 (500 MHz for ^{1}H and 125 MHz for ^{13}C) and Agilent 400-MR (400 MHz for ^{1}H and 100 MHz for ^{13}C) spectrometers. ^{13}C NMR for the polymers were mainly recorded by Bruker (600 MHz for ^{1}H and 150 MHz for ^{13}C) spectrometers in the National Instrumentation Center for Environmental Management (NICEM) at SNU. High resolution mass spectroscopy (HRMS) analyses were performed by the ultrahigh resolution ESI Q-TOF mass spectrometer (Bruker, Germany) in the Sogang Centre for Research Facilities. Size exclusion chromatography (SEC) analyses were carried out with Waters system (1515 pump, 2414 refractive index detector) and Shodex GPC LF-804 column eluted with THF (GPC grade, Honeywell Burdick & Jackson®) and filtered with a 0.2 µm PTFE filter (Whatman®). Flow rate was 1.0 mL/min and temperature of column was maintained at 35 °C.

2. Experimental procedures for the preparation of the monomers

Ru1,[1] **Ru2**,[2] **M1**, **M3-M6**, **M8-M11**[3] were prepared by literature methods.

M2 (*tert*-butyl 2-(prop-2-yn-1-yl)-2-(2-((triisopropylsilyl)oxy)propan-2-yl)pent-4-ynoate)

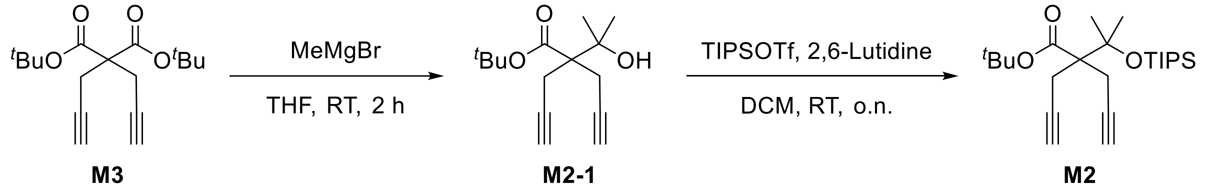

To a solution of **M3** (880 mg, 3.0 mmol) in THF (9 ml), methylmagnesium bromide (3 M in ether, 12 mmol, 4 ml) was slowly added at 0 °C. and The mixture was stirred for 2 hr at room temperature then quenched by saturated NH_4Cl aqueous solution at 0 °C. The organic layer was washed with NaCl aqueous solution, extracted by EtOAc, dried with $MgSO_4$, and concentrated. The product was purified by flash column chromatography on silica gel (EtOAc:Hexane=1:20) to afford **M2-1** as a colorless liquid (510 mg, 68% yield). ^{1}H-NMR (500 MHz, $CDCl_3$): δ 3.28 (s, 1H), 2.83 (q, J = 61.0, 17.2 Hz, 4H), 2.05 (s, 2H), 1.49 (s, 9H), 1.31 (s, 6H).; ^{13}C-NMR (125MHz, $CDCl_3$): δ 172.7, 82.9, 81.9, 74.1, 71.3, 56.2, 28.1, 26.6, 21.8.; HR-MS (ESI) m/z for $C_{15}H_{22}NaO_3$ $[M+Na]^+$, calcd. 273.1461, found: 273.1460.

To a solution of **M2-1** (750mg, 3.0 mmol) in DCM (9 mL), 2,6-lutidine (1.4 mL, 12 mmol) was added and the mixture was cooled down to 0 °C, followed by the addition of triisopropylsilyl trifluoromethanesulfonate (1.1 mL, 6 mmol). After stirring overnight at room temperature, the mixture was quenched by saturated NH_4Cl aqueous solution. The organic layer was washed with NaCl aqueous solution, extracted by EtOAc, dried with $MgSO_4$, and concentrated. The product was purified by flash column chromatography on silica gel (hexane only) to afford **M2** as a colorless liquid (650 mg, 53% yield). ^{1}H-NMR (400 MHz, $CDCl_3$): δ 2.83 (q, J = 16.8 Hz, 4H), 1.98 (s, 2H), 1.47 (s, 9H), 1.44 (s, 6H), 1.09 (s, 21H).; ^{13}C-NMR (150MHz, $CDCl_3$): δ 171.8, 82. 7, 81.7, 77.0, 70.4, 57.6, 28.1, 22.4, 18.6, 13.8.; HR-MS (ESI): m/z for $C_{24}H_{42}NaO_3Si$, $[M+Na]^+$, calcd. 429.2795, found: 429.2797.

M7 (di(adamantan-1-yl) 2,2-di(prop-2-yn-1-yl)malonate)

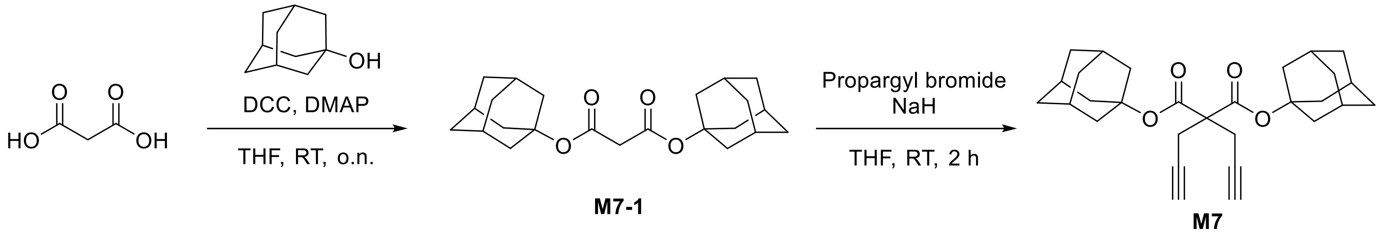

Malonic acid (310 mg, 3.0 mmol) and 1-adamantanol (1.0 g, 6.6 mmol) were solvated in THF (15 ml). A mixture of *N,N'*-dicyclohexylcarbodiimide (1.4 g, 6.6 mmol) and 4-dimethylaminopyridine (36 mg, 0.30 mmol) in THF (15 mL) was slowly added at 0 °C. The mixture was stirred overnight at room temperature then quenched by acetic acid. After partially removing dicyclohexylurea (generated as a byproduct) by filtering, the organic layer was washed with water and extracted by DCM, dried with $MgSO_4$, and concentrated. The product was purified by flash column chromatography on silica gel (EtOAc:Hexane = 1:10) to afford **M7-1** as white solid (740 mg, 66% yield). ^{1}H-NMR (500 MHz, $CDCl_3$): δ 3.18 (s, 2H), 2.17 (s, 6H), 2.13 (s, 12H), 1.66 (s, 12H). ^{13}C-NMR (150 MHz, $CDCl_3$): δ 166.09, 81.83, 44.78, 41.33, 36.28, 30.97. HR-MS (ESI) m/z for $C_{23}H_{32}NaO_4$ $[M+Na]^+$, calcd. 395.2193, found: 395.2196.

Sodium hydride (60%, dispersion in mineral oil) (88 mg, 2.2 mmol) in THF (2 mL) was prepared at 0 ℃ in RBF purged with argon and a solution of **M7-1** (370 g, 1.0 mmol) in THF (1 mL) was added drop-wisely. After 10 minutes of stirring, propargyl bromide (80 wt%, in toluene) (0.50 mL, 2.5 mmol) was added and stirred for 2 hr. The reaction was quenched by adding NH_4Cl aqueous solution and the organic layer was extracted with EtOAc, dried with $MgSO_4$, and concentrated. The product was purified by flash column chromatography on silica gel (EtOAc:Hexane = 1:30) to afford **M7** as a white solid (410 mg, 91% yield). ^{1}H-NMR (500 MHz, $CDCl_3$): δ 2.88 (d, J = 2.6 Hz, 4H), 2.18 (s, 6H), 2.11 (d, J = 2.9 Hz, 12H), 2.02 (t, 2H), 1.66 (s, 12H). ^{13}C-NMR (150 MHz, $CDCl_3$): δ 167.7, 82.3, 79.2, 71.5, 57.2, 41.2, 36.3, 31.0, 22.5.; HR-MS (ESI) m/z for $C_{29}H_{36}NaO_4$ $[M+Na]^+$, calcd. 471.2506, found: 471.2509.

3. General procedure for the cyclopolymerization

A 5-mL sized sealed vial with septum was flame dried and charged with monomer and a magnetic bar. The vial was purged with argon four times, and degassed anhydrous DCM was added. After the Ar-purged catalyst (**Ru1** and **Ru2**) and pyridine additive in another 5-mL vial were dissolved in DCM, the solution was rapidly injected to the monomer solution at experimental temperature under vigorous stirring. The reaction was quenched by excess ethyl vinyl ether after desired reaction time, and partially precipitated in hexane or methanol at -78 °C, remaining small amount of crude mixture solution (~10%). Obtained solid was filtered and dried in vacuo. Monomer conversion was calculated from the ^{1}H NMR spectrum of the remaining crude mixture.

4. Calculation of the regioselectivity for P5 using ^{1}H and ^{13}C NMR

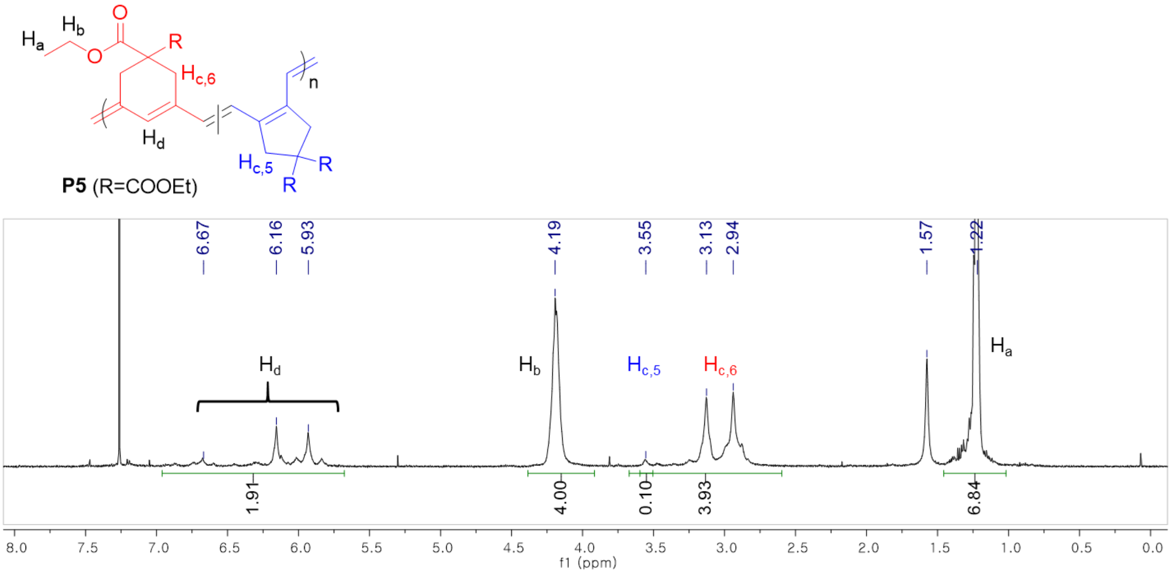

<Figure S1. ^{1}H NMR spectrum of the crude mixture of entry 1 in Table 2>

$$\text{Composition of five-membered ring} = \frac{2*H_{c,5}}{H_{\text{originated from propargylic}}}$$

e.g. (entry 1 in Table 2)

$$\text{Composition of five-membered ring} = \frac{2*0.10}{3.93} = 0.051 \quad (\therefore \beta\text{-selectivity=95\%})$$

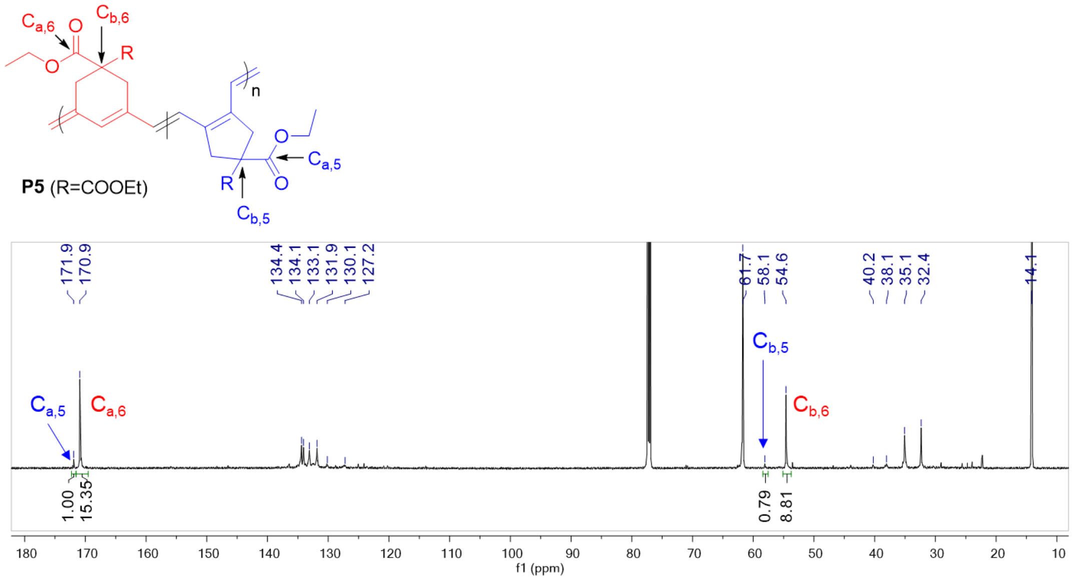

<Figure S2. ^{13}C NMR spectrum of **P5** from entry 1 in Table 3>

$$\text{Composition of five-membered ring} = \frac{C_{a,5}}{C_{a,5}+C_{a,6}} \; or \; \frac{C_{b,5}}{C_{b,5}+C_{b,6}}$$

e.g. (entry 1 in Table 3)

$$\text{Composition of five-membered ring} = \frac{1}{16.35} = 0.061 \quad (\therefore \beta\text{-selectivity=94\%})$$

5. *In situ* NMR experiment: procedure and data

Initiation experiment of Ru1

To an NMR tube was added a solution of **Ru1** (2.3 mg, 0.003 mmol) in 0.6 mL DCM-*d2*. The tube was then sealed with a rubber septum, taken out of the glovebox, and placed in a dry ice/acetone bath. Butyl vinyl ether (12 µL, 0.090 mmol) was injected into the tube, and the reaction was monitored by observing the disappearance of the benzylidene signal by ^{1}H NMR using an array at the appropriate temperature.

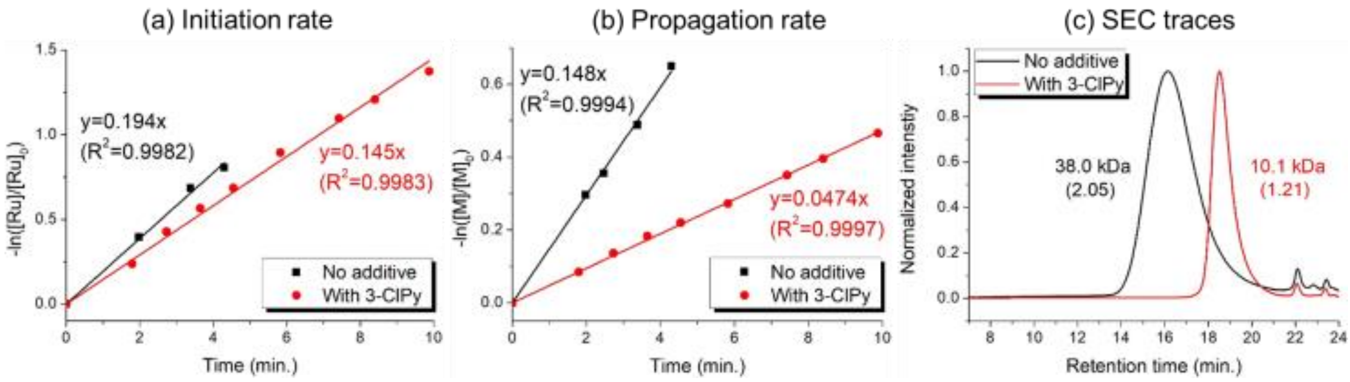

<Figure S3. Plots of (a) -ln([Ru]/[Ru]$_0$) and (b) -ln([M]/[M]$_0$) vs. time for the CP of **M2** for M/I=20, with and without 3-ClPy, and (c) SEC traces of the resulting polymers>

Kinetic experiments using Ru1 or **Ru2**

Ru1 or **Ru2** (0.003 mmol, 1 eq) and hexamethyldisilane (internal standard, 3 µl) were dissolved in DCM-*d2* (400 µL). Initial benzylidene was measured by integral ratio of **Ru1** or **Ru2** to hexamethyldisilane in ^{1}H NMR spectrum. (After the addition of 4-7 eq of the pyridine additive,) Monomer (0.06 mmol, 20 eq) solution in DCM-*d2* (200 µl) was added to the catalyst solution and mixed by shaking the NMR tube for 5 seconds. The reaction was monitored by ^{1}H NMR over time. The k_i or k_p values were obtained from the slope of linear – ln [**Ru**]/[**Ru**]$_0$ or – ln [M]/[M]$_0$ vs. time graphs, respectively.

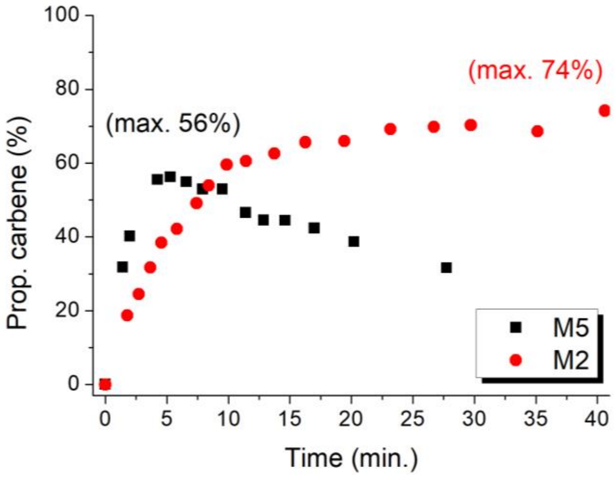

<Figure S4. Plot of the propagating carbene vs. reaction time for **M5** and **M2** using **Ru1** under 3-ClPy as an additive>

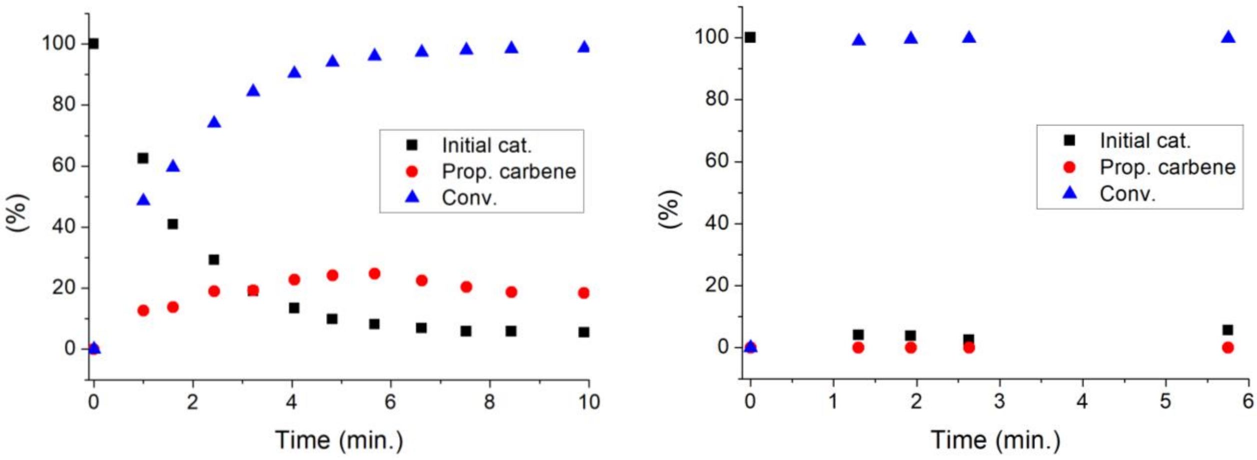

<Figure S5. Plot of the conversion and initial Ru catalysts vs. reaction time for **M5** using **Ru1** (left) and **Ru2** (right) under 3,5-Cl_2Py as an additive>

6. SEC traces of the polymers

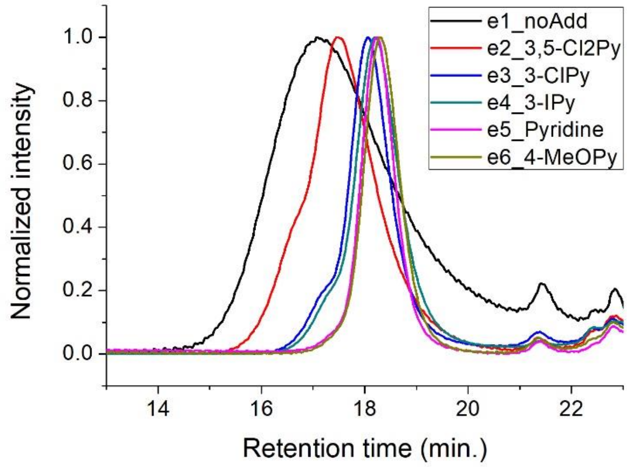

<Figure S6. SEC traces of **P5**s in Table 2>

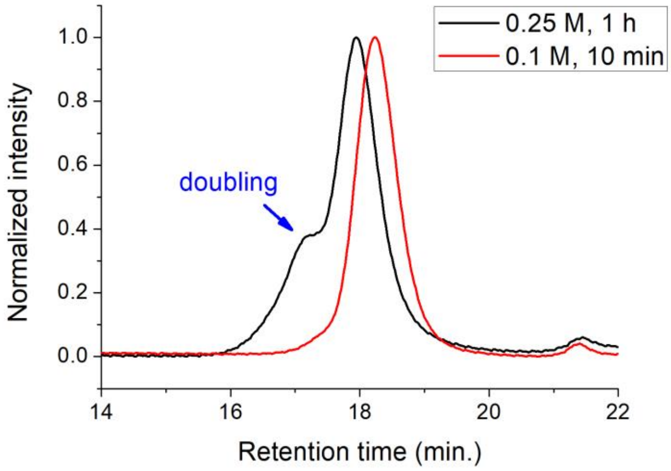

<Figure S7. SEC traces of **P5**s synthesized in different conditions>

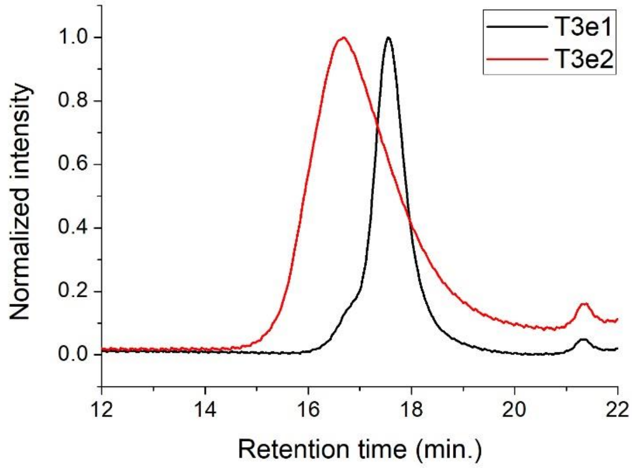

<Figure S8. SEC traces of **P5**s in Table 3, entries 1 and 2>

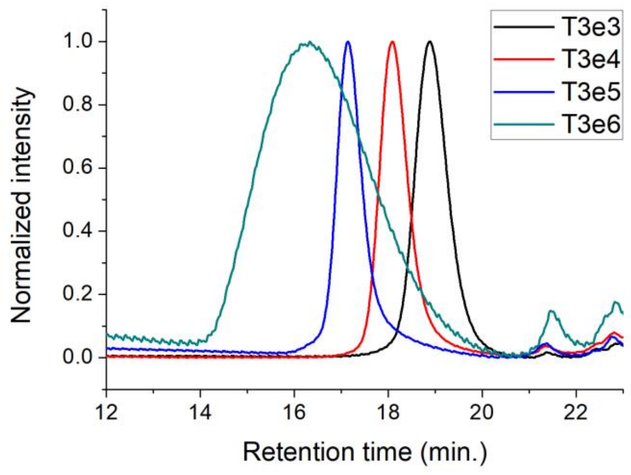

<Figure S9. SEC traces of **P6**s in Table 3, entries 3-6>

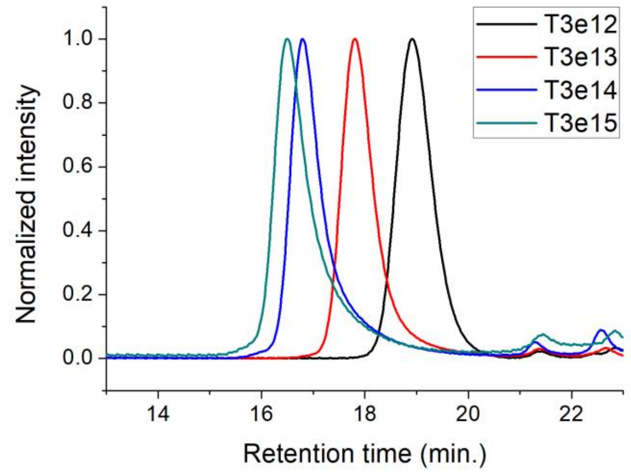

<Figure S10. SEC traces of **P7**s in Table 3, entries 12-15>

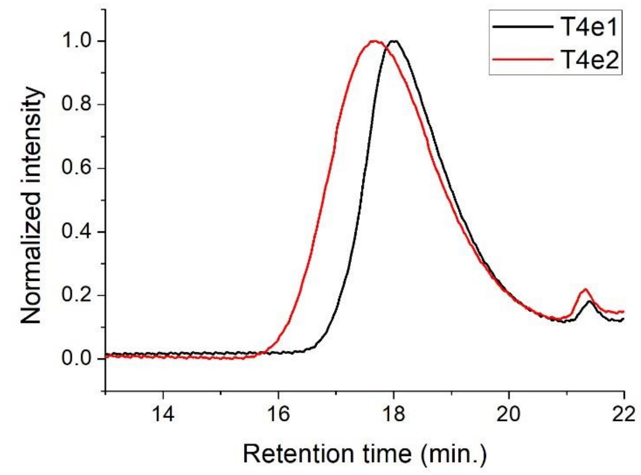

<Figure S11. SEC traces of **P8**s in Table 4, entries 1 and 2>

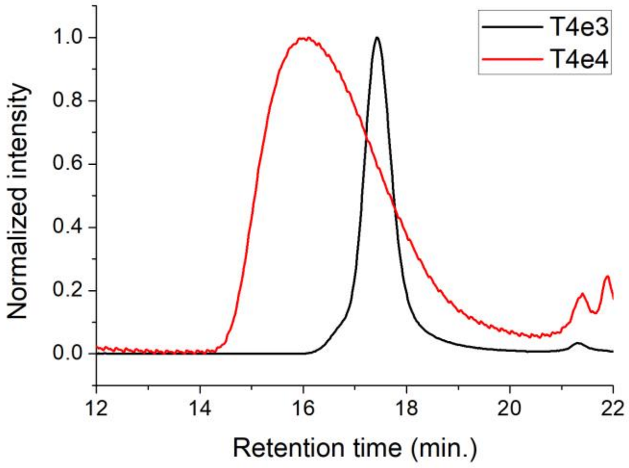

<Figure S12. SEC traces of **P9**s in Table 4, entries 3 and 4>

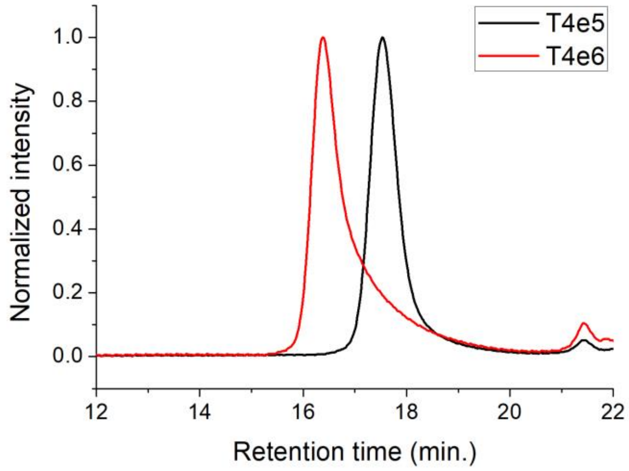

<Figure S13. SEC traces of **P4**s in Table 4, entries 5 and 6>

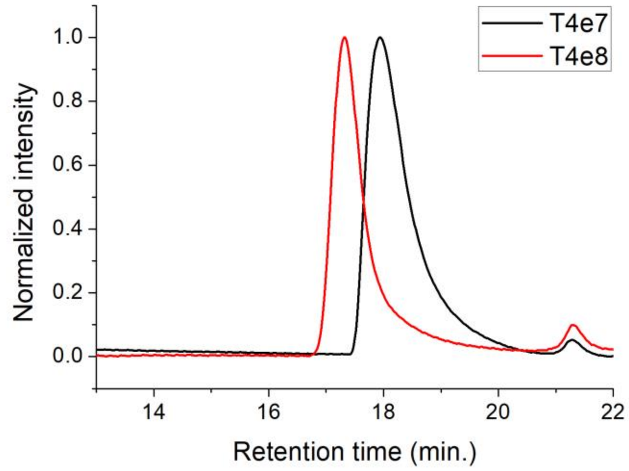

<Figure S14. SEC traces of **P1**s in Table 4, entries 7 and 8>

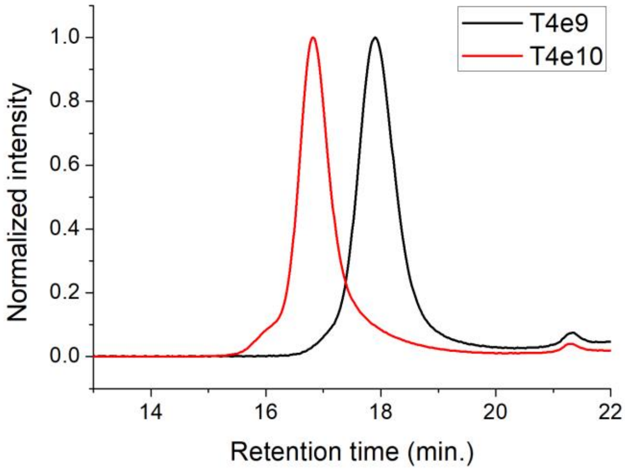

<Figure S15. SEC traces of **P10**s in Table 4, entries 9 and 10>

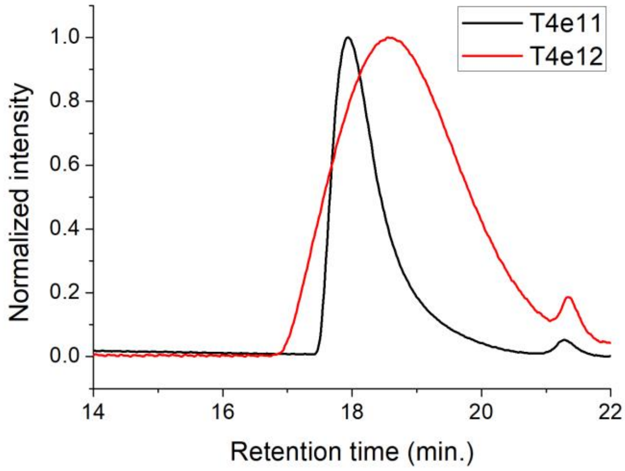

<Figure S16. SEC traces of **P11**s in Table 4, entries 11 and 12>

7. ^{1}H and ^{13}C NMR characterization of polymers

The ^{1}H NMR and ^{13}C NMR of **P1**, **P3**-**6**, **P8**-**11** are described in the literature.[3]

P2
^{1}H (500 MHz, $CDCl_3$): δ 7.04 – 5.62 (br m, 2H), 3.68 – 2.40 (br m, 4H), 1.42 (br s, 6H), 1.29 (br s, 9H), 1.11 (br s, 21H); ^{13}C (150 MHz, $CDCl_3$): δ 172.9, 140.3, 137.5, 136.9, 134.0, 131.4, 80.3, 75.9, 57.8, 33.2, 30.6, 27.8, 18.8, 13.8.

P2$_{15}$-*b*-P3$_{15}$
^{1}H (500 MHz, $CDCl_3$): δ 7.04 – 5.62 (br m, 4H), 3.68 – 2.40 (br m, 8H), 1.42 (br s, 24H), 1.29 (br s, 9H), 1.11 (br s, 21H); ^{13}C (150 MHz, $CDCl_3$): δ 172.9, 170.1, 137.4, 134.6, 133.2, 131.7, 128.1, 81.5, 80.3, 75.92, 57.8, 55.6, 35.3, 32.5, 30.7, 28.0, 18.8, 13.8.

P2$_{15}$-*b*-P4$_{15}$
^{1}H (500 MHz, $CDCl_3$): δ 7.04 – 5.62 (br m, 4H), 3.88 – 2.07 (br m, 12H), 1.39 (br s, 6H), 1.29 (br s, 9H), 1.11 (br s, 21H), 1.04 (br s, 42H); ^{13}C (150 MHz, $CDCl_3$): δ 172.9, 140.4, 137.4, 135.9, 133.4, 132.3, 80.3, 75.9, 66.0, 57.8, 41.3, 33.6, 27.8, 18.8, 18.3, 13.8, 12.2.

P7
^{1}H (500 MHz, $CDCl_3$): δ 6.90 – 5.70 (br m, 2H), 3.50 – 2.59 (br m, 4H), 2.14 (br s, 6H), 2.08 (br s, 12H), 1.64 (br s, 12H); ^{13}C (150 MHz, $CDCl_3$): δ 169.9, 134.8, 134.3, 133.4, 131.9, 81.4, 55.7, 41.1, 36.3, 31.0.

P9$_{15}$-*b*-P3$_{15}$
^{1}H (500 MHz, $CDCl_3$): δ 6.92 – 5.66 (br m, 2H), 3.39 (br s, 4H), 3.16 – 2.06 (br m, 8H), 1.42 (br s, 18H), 0.88 (br s, 18H), 0.00 (br s, 12H); ^{13}C (150 MHz, $CDCl_3$): δ 170.2, 135.6, 134.7, 134.4, 133.4, 132.0, 128.0, 81.4, 65.7, 65.3, 55.7, 47.7, 40.6, 35.3, 33.5, 32.4, 30.9, 28.0, 26.1, 18.4, -5.4.

P3$_{15}$-*b*-P7$_{15}$
^{1}H (500 MHz, $CDCl_3$): δ 6.90 – 5.70 (br m, 4H), 3.50 – 2.59 (br m, 8H), 2.14 (br s, 6H), 2.08 (br s, 12H), 1.64 (br s, 12H), 1.42 (br s, 18H); ^{13}C (150 MHz, $CDCl_3$): δ 170.1, 169.9, 134.7, 134.4, 133.4, 132.0, 81.4, 55.7, 55.6, 41.2, 36.3, 35.3, 32.4, 31.0, 28.0.

P3$_{15}$-*b*-P7$_{15}$-*b*-P11$_{15}$
^{1}H (500 MHz, $CDCl_3$): δ 6.90 – 5.63 (br m, 6H), 3.50 – 2.46 (br m, 20H), 2.14 (br s, 6H), 2.08 (br s, 12H), 1.64 (br s, 12H), 1.42 (br s, 18H), 1.08 (br s, 12H); ^{13}C (150 MHz, $CDCl_3$): δ 170.1, 169.9, 134.9, 134.3, 133.4, 132.0, 81.4, 55.7, 55.6, 53.6, 41.1, 36.3, 35.3, 32.4, 30.9, 27.9, 14.0, 12.9.

8. ^{1}H and ^{13}C NMR spectra of the polymers

^{13}C NMR spectra were used for the determination of the ratio between five- and six-ring on the polymer backbone.

<**P2** from Table 1>

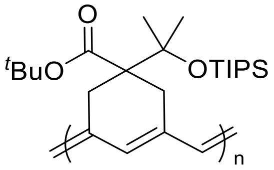

^{1}H NMR (500 MHz, $CDCl_3$)

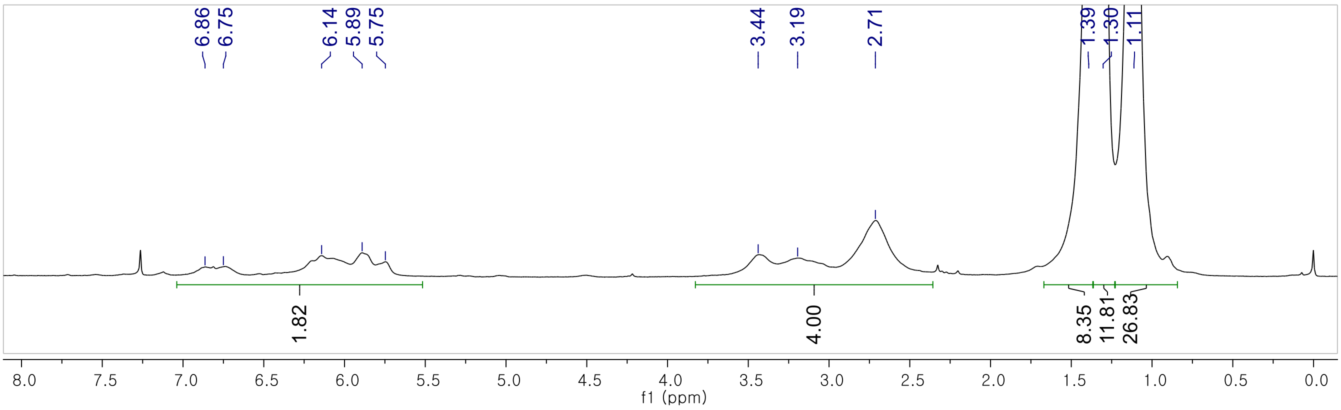

^{13}C NMR (150 MHz, $CDCl_3$)

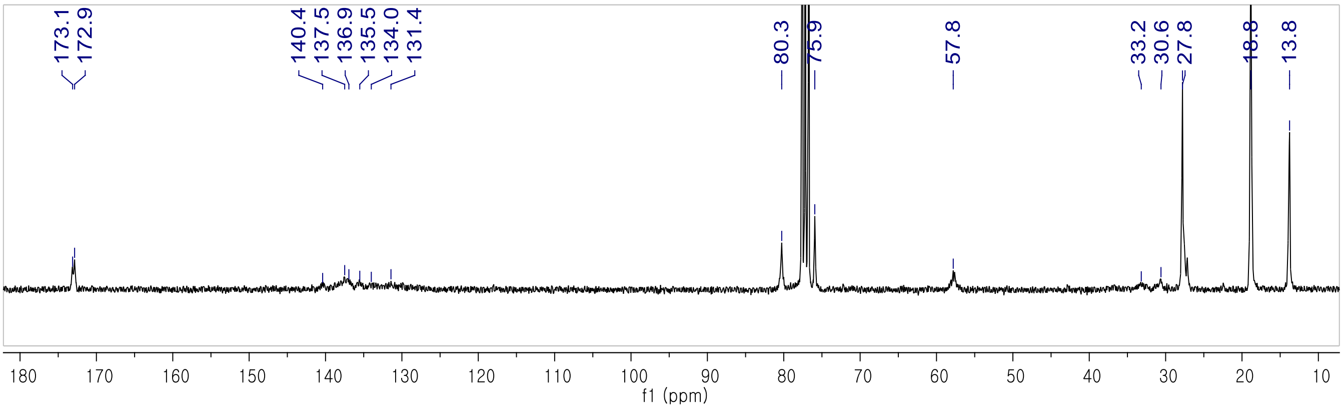

<**P2**-*b*-**P3** from Scheme 2>

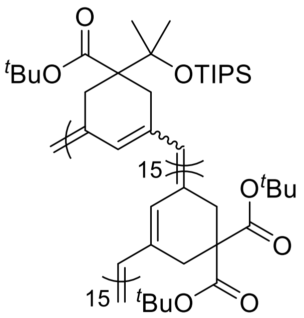

^{1}H NMR (500 MHz, CDCl$_3$)

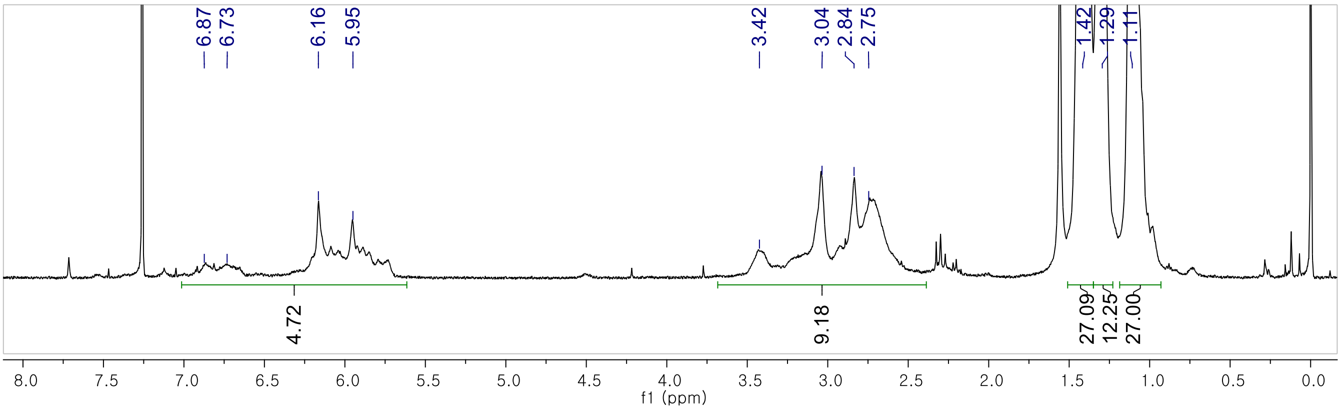

^{13}C NMR (150 MHz, CDCl$_3$)

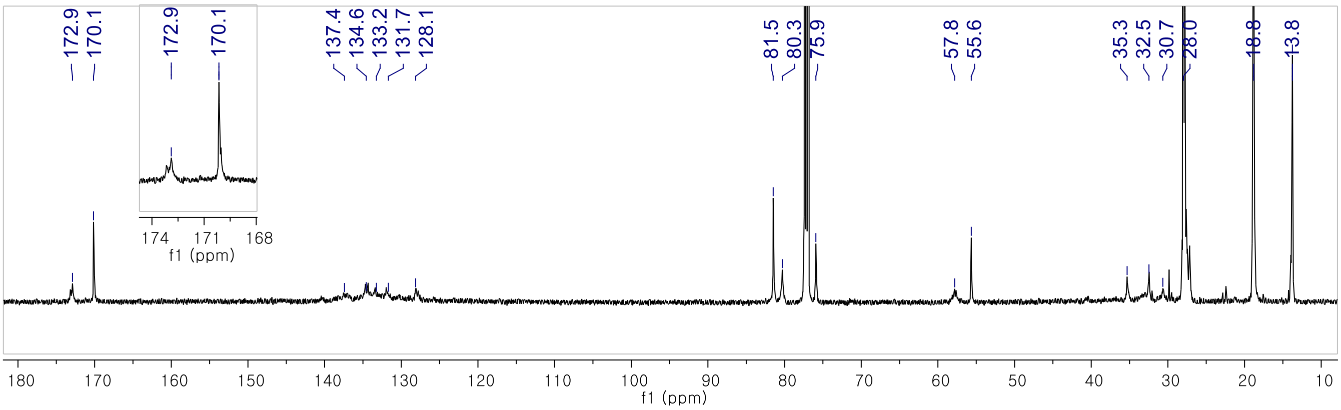

<**P2**-*b*-**P4** from Scheme 2>

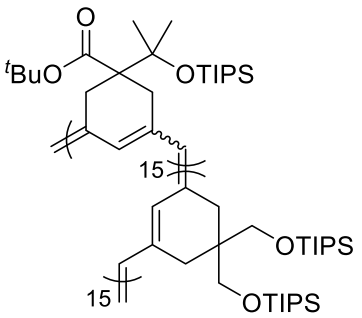

^{1}H NMR (500 MHz, $CDCl_3$)

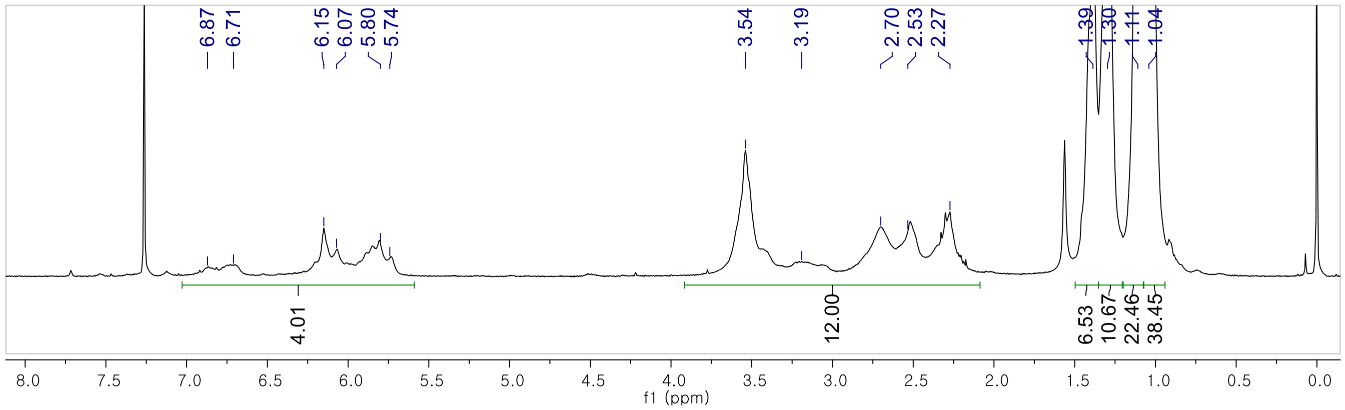

^{13}C NMR (150 MHz, $CDCl_3$)

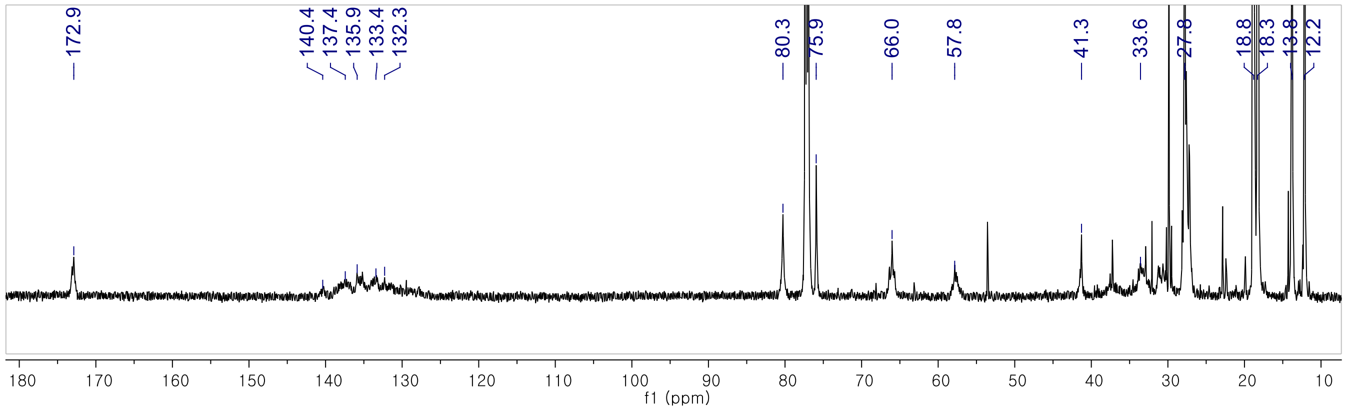

<**P5** from Table 3, entry 1>

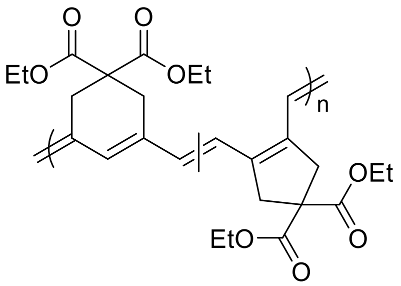

^{1}H NMR (500 MHz, $CDCl_3$)

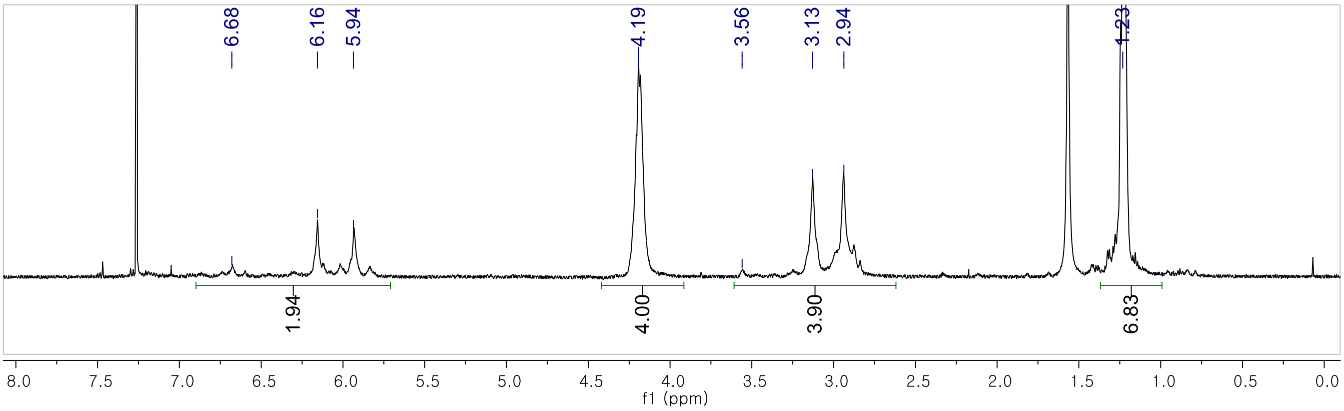

^{13}C NMR (150 MHz, $CDCl_3$)

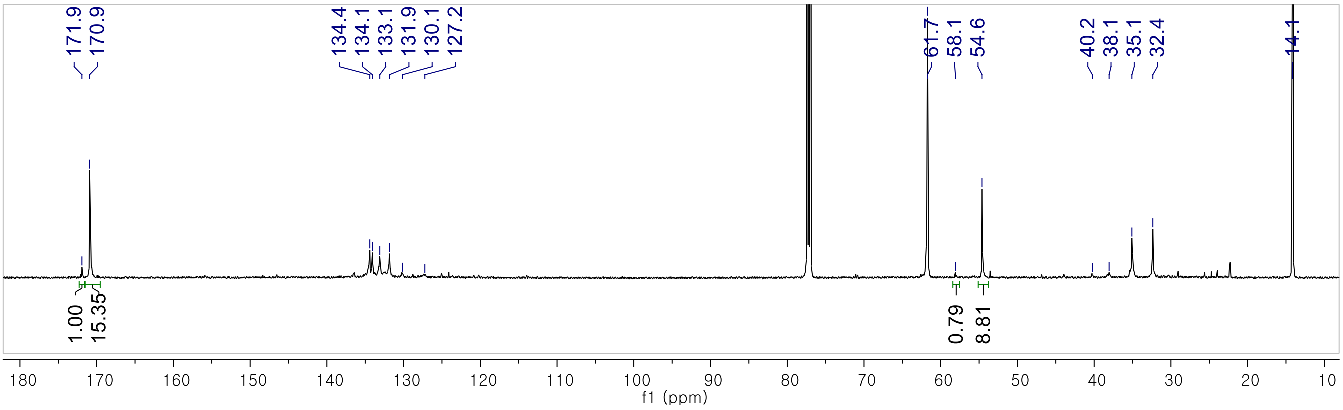

< **P6** from Table 3, entry 5>

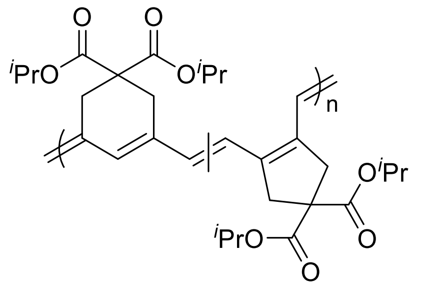

^{1}H NMR (500 MHz, $CDCl_3$)

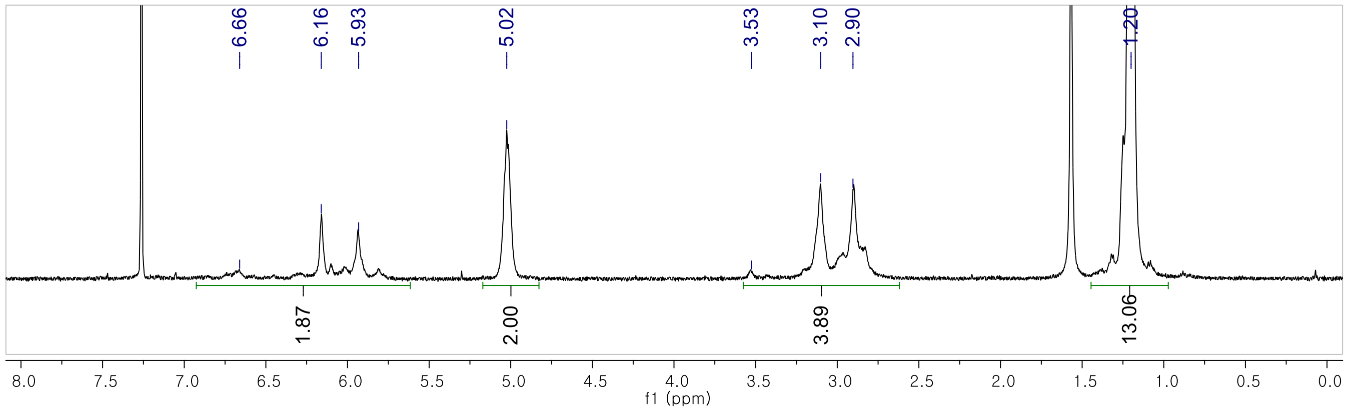

^{13}C NMR (150 MHz, $CDCl_3$)

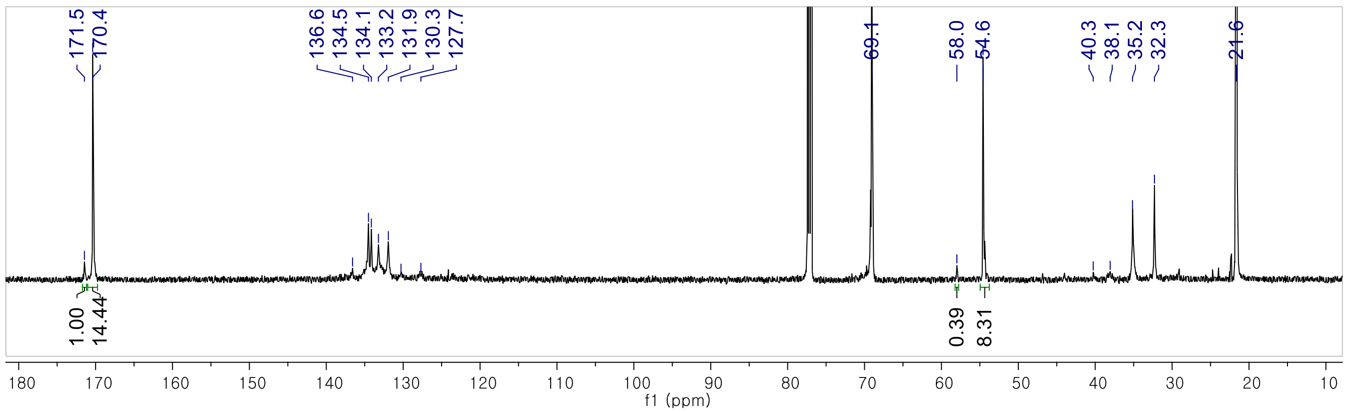

<**P3** from Table 3, entry 9>

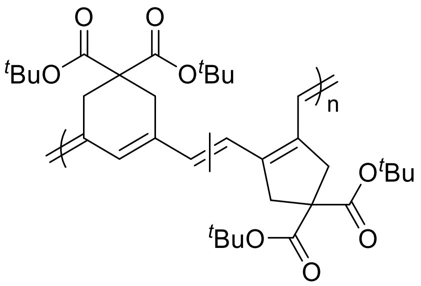

^{1}H NMR (500 MHz, $CDCl_3$)

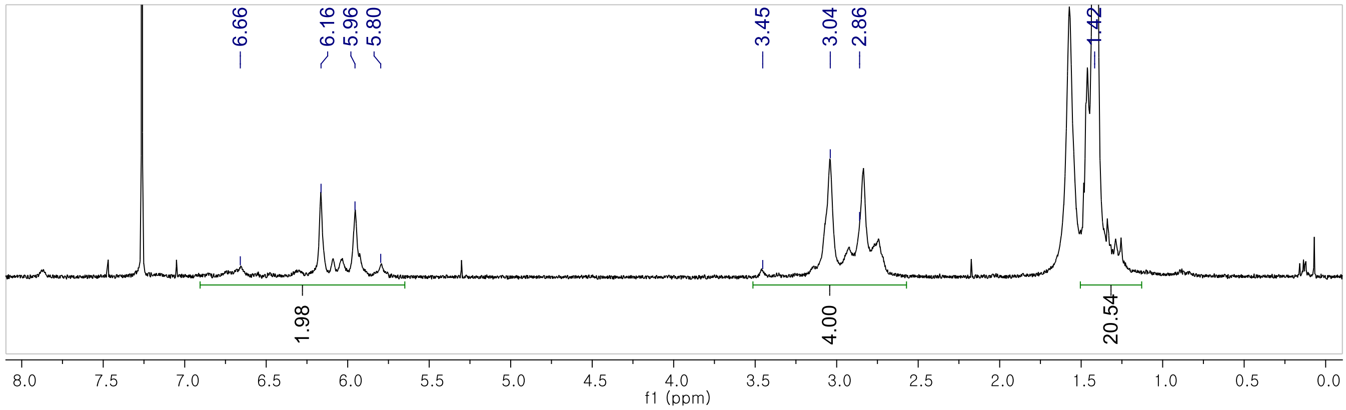

^{13}C NMR (150 MHz, $CDCl_3$)

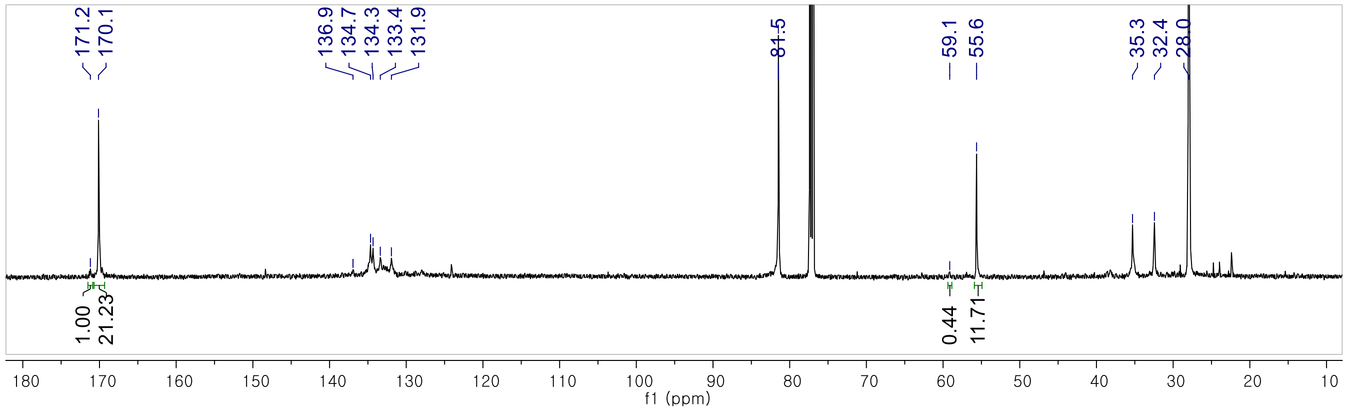

<**P7** from Table 3, entry 14>

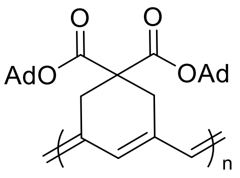

^{1}H NMR (500 MHz, $CDCl_3$)

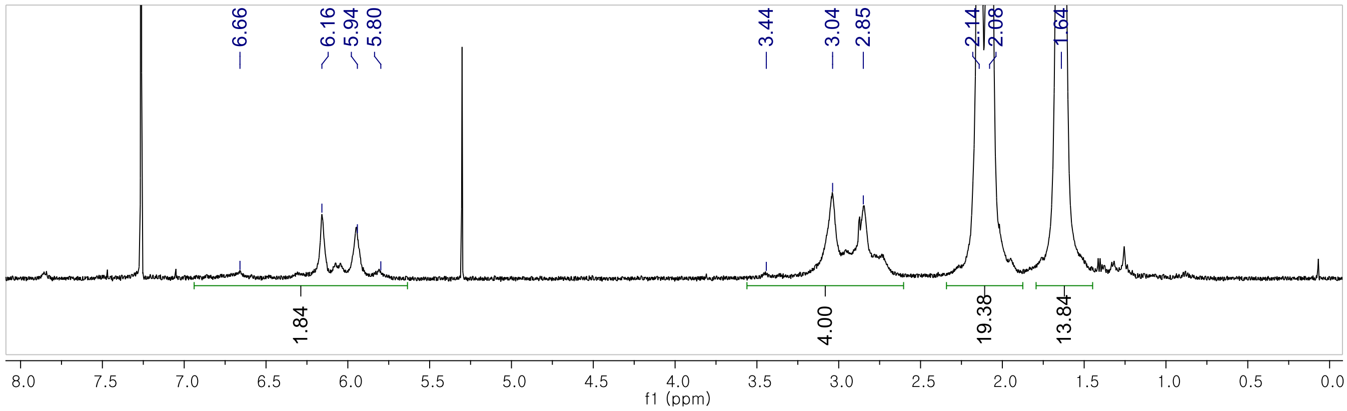

^{13}C NMR (150 MHz, $CDCl_3$)

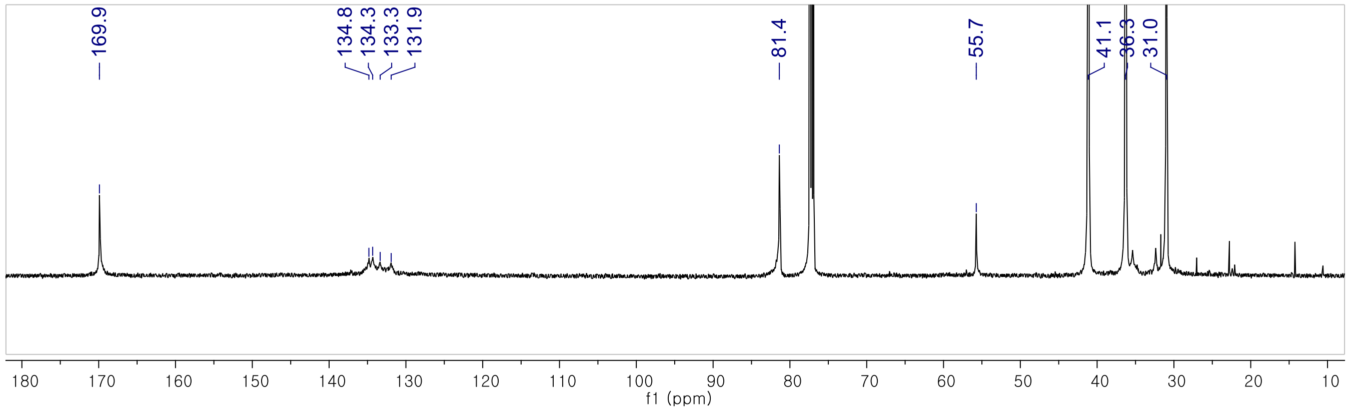

<**P8** from Table 4, entry 1>

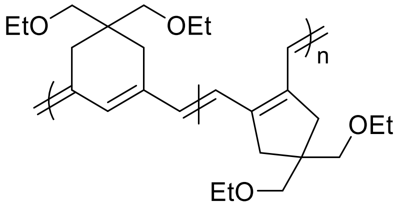

^{1}H NMR (500 MHz, $CDCl_3$)

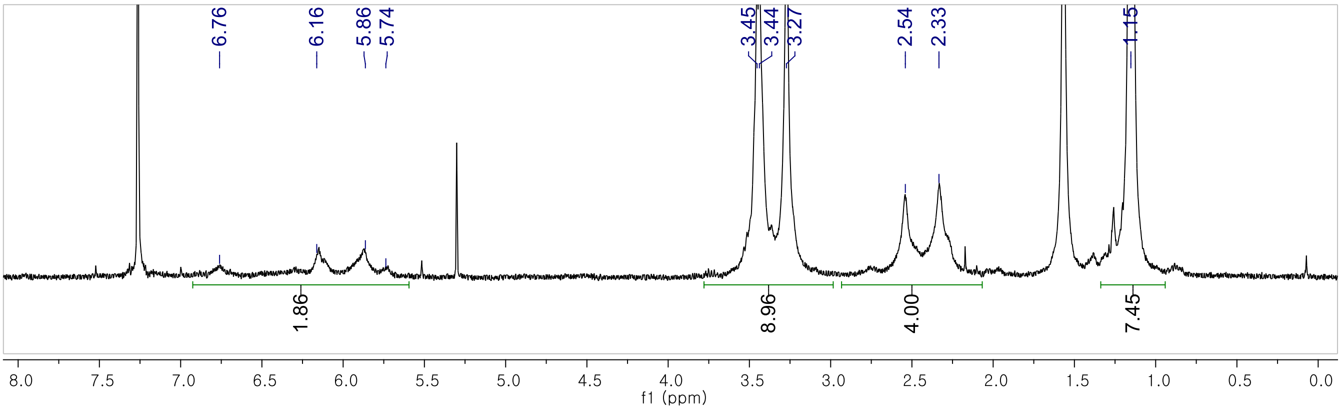

^{13}C NMR (150 MHz, $CDCl_3$)

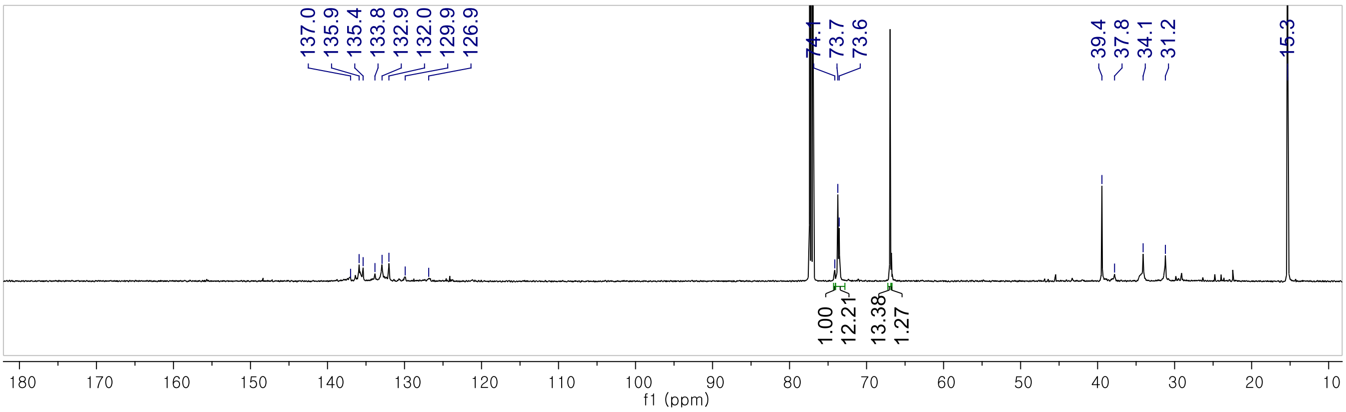

<**P9** from Table 4, entry 4>

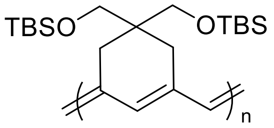

^{1}H NMR (500 MHz, $CDCl_3$)

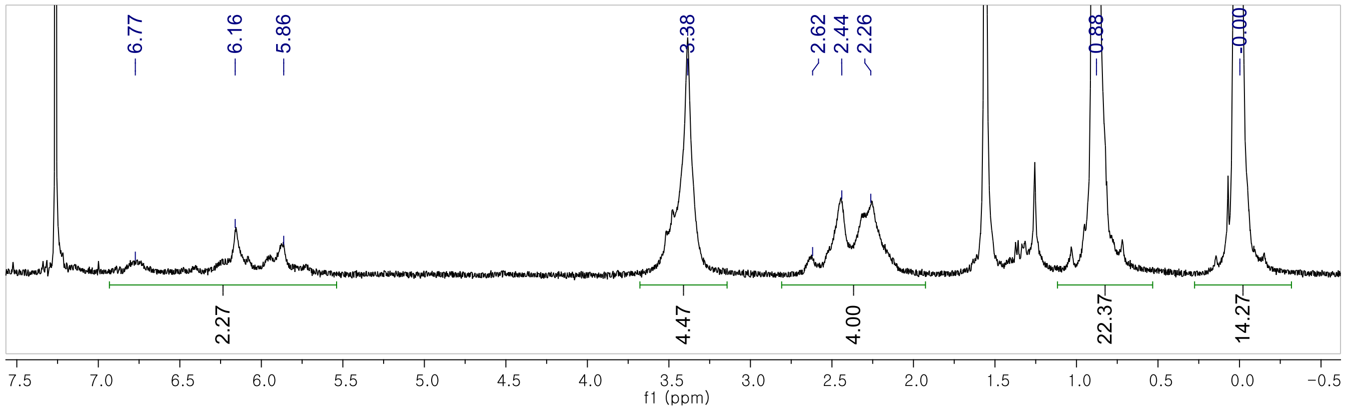

^{13}C NMR (150 MHz, $CDCl_3$)

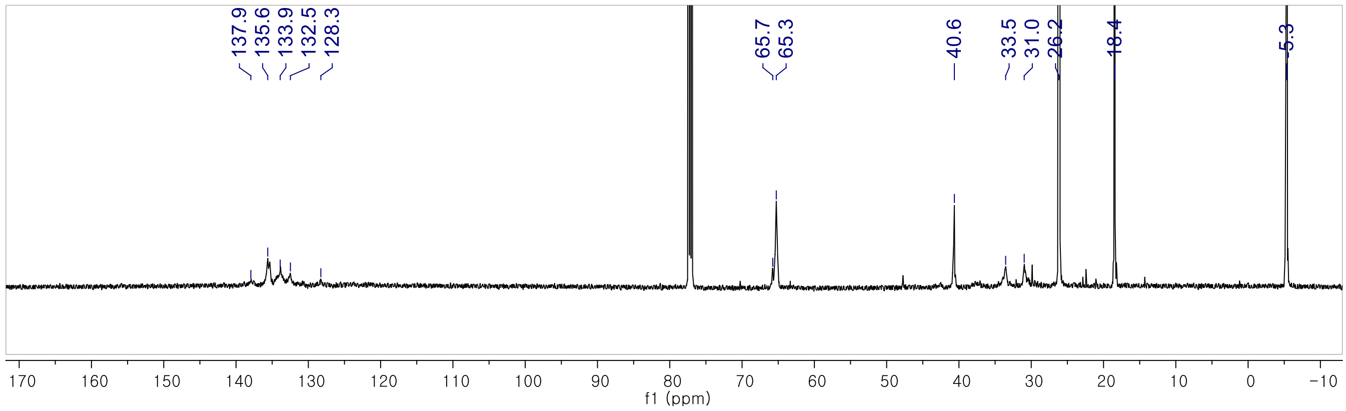

<**P4** from Table 4, entry 6>

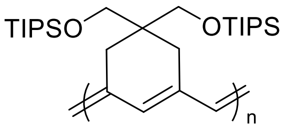

^{1}H NMR (500 MHz, $CDCl_3$)

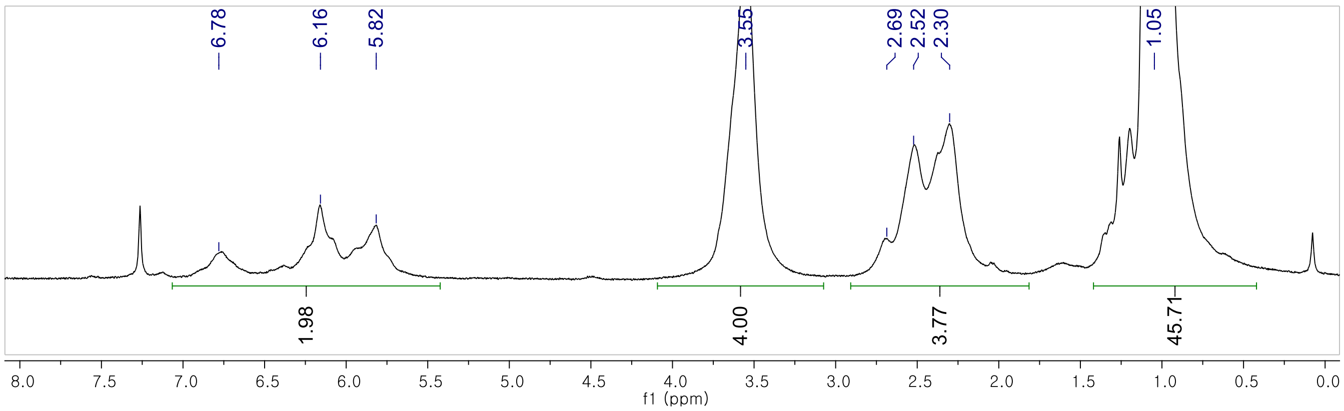

^{13}C NMR (150 MHz, $CDCl_3$)

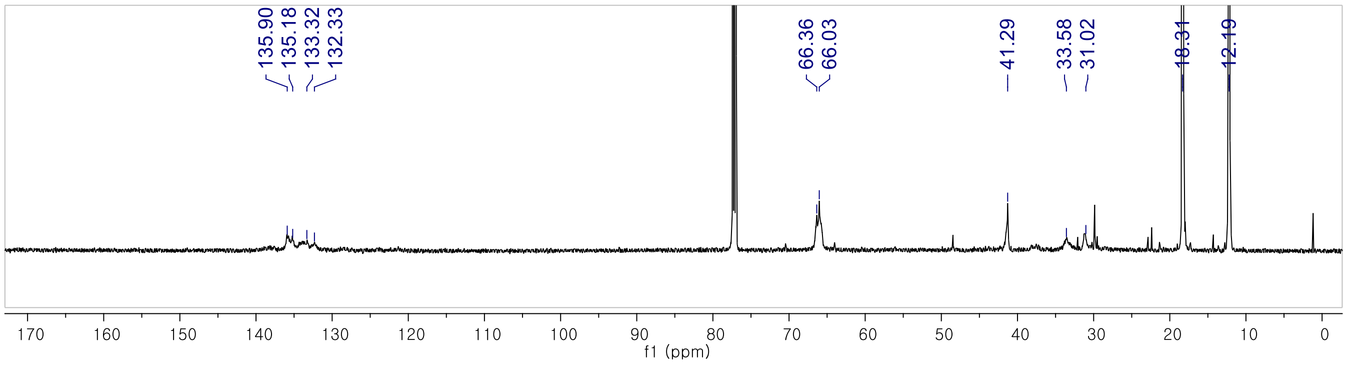

<**P1** from Table 4 entry 7>

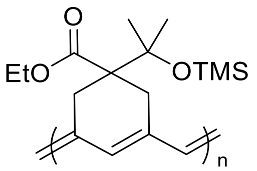

^{1}H NMR (500 MHz, $CDCl_3$)

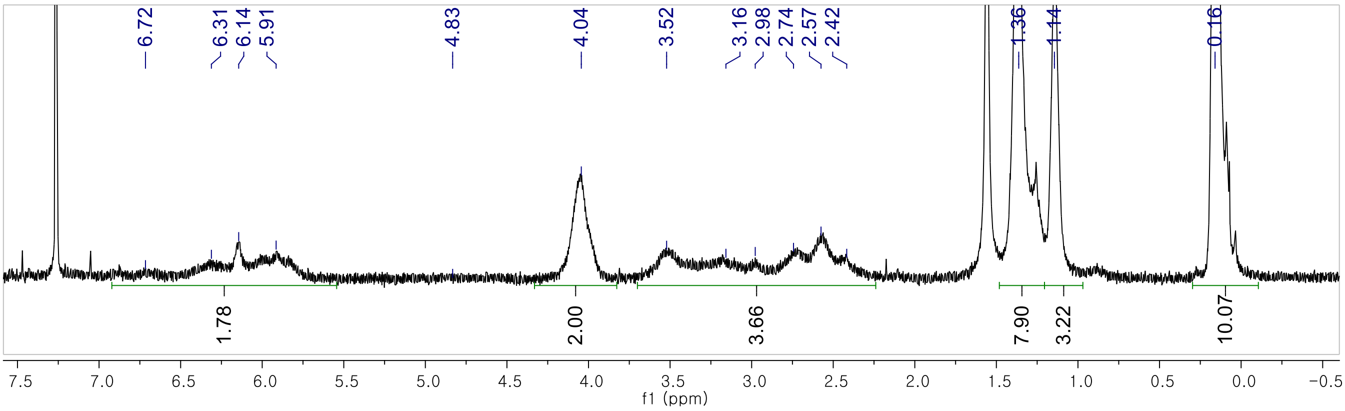

^{13}C NMR (150 MHz, $CDCl_3$)

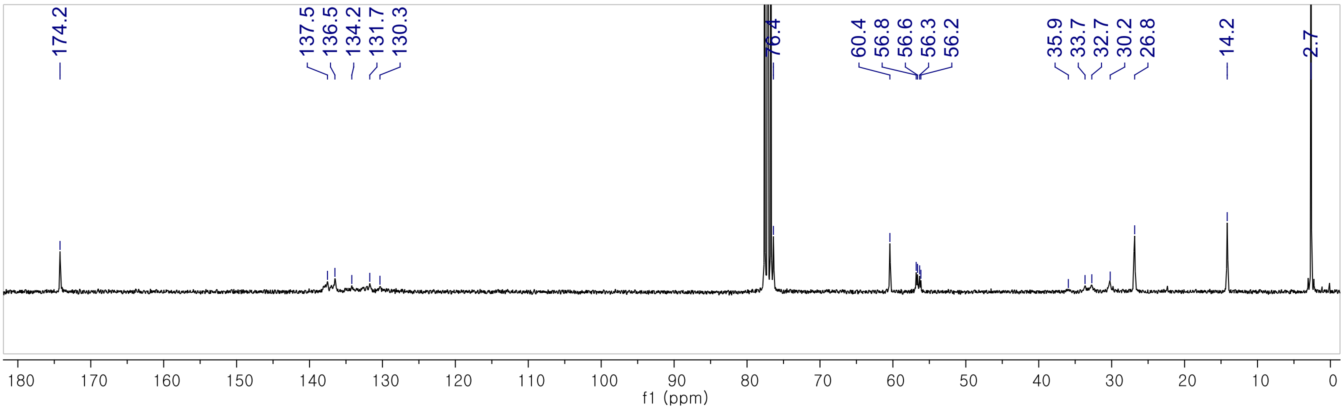

<**P10** from Table 4, entry 10>

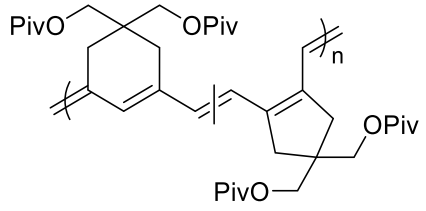

^{1}H NMR (500 MHz, $CDCl_3$)

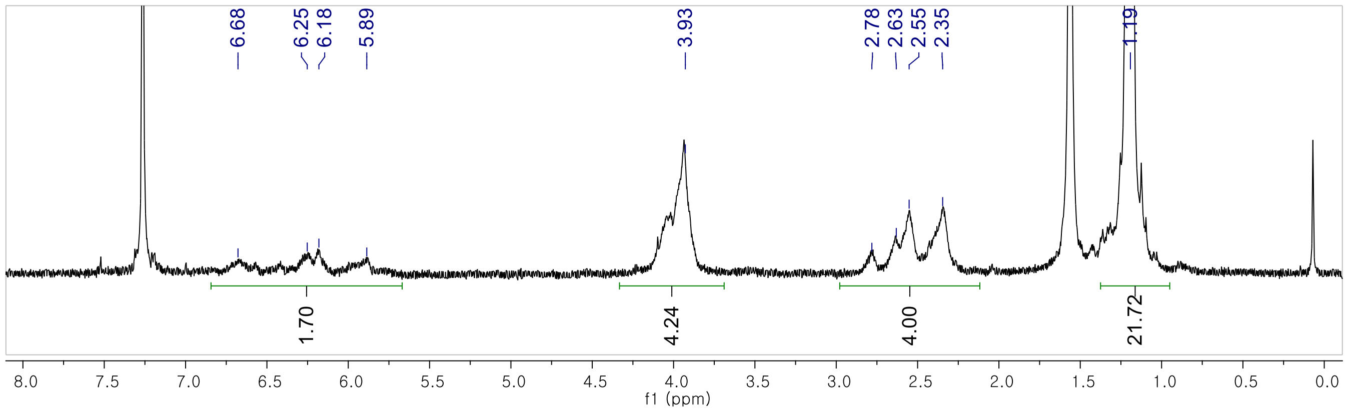

^{13}C NMR (150 MHz, $CDCl_3$)

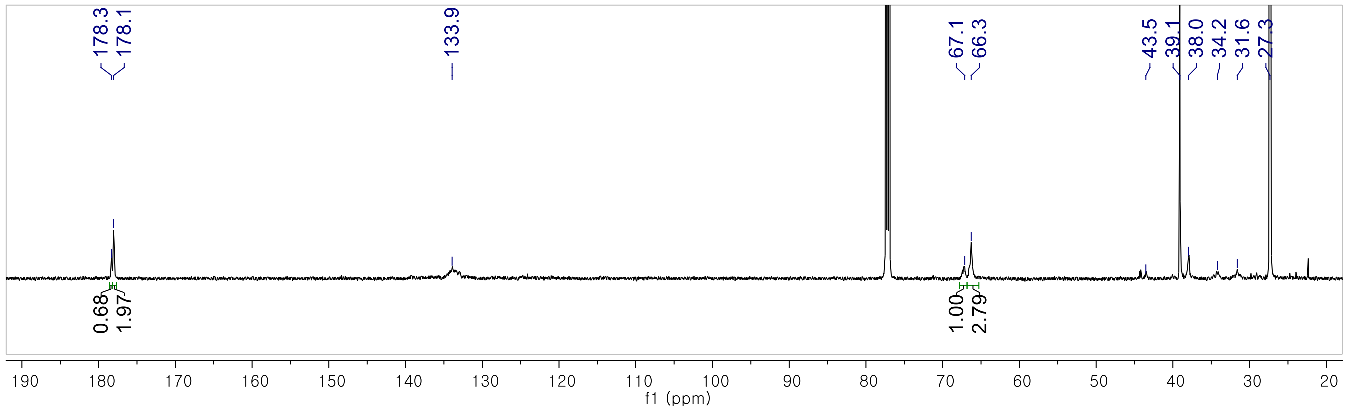

<**P11** from Table 4, entry 11>

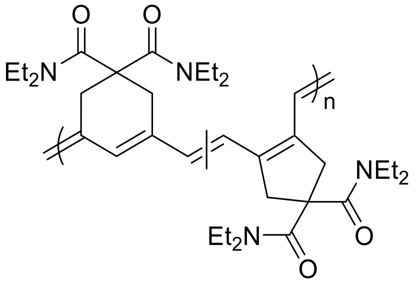

^{1}H NMR (500 MHz, $CDCl_3$)

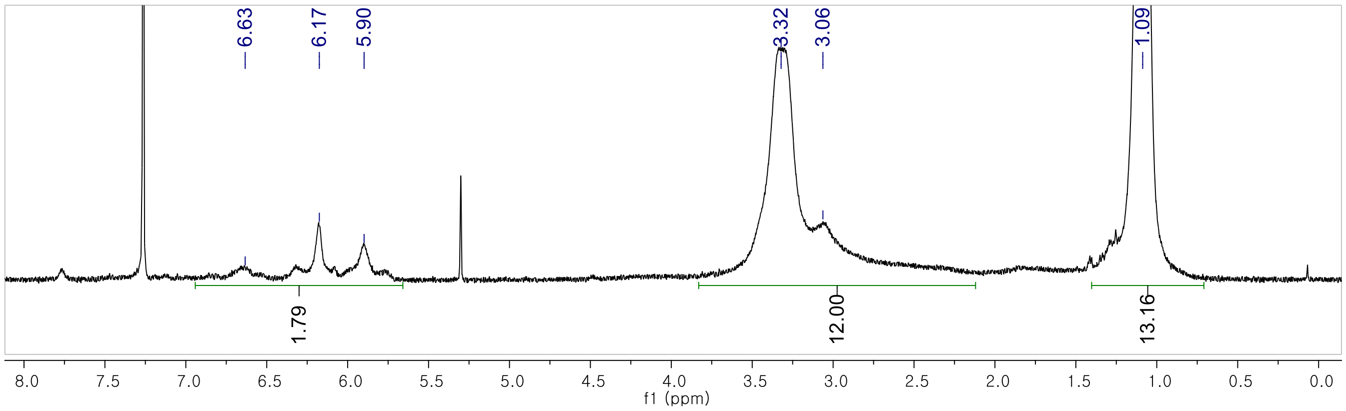

^{13}C NMR (150 MHz, $CDCl_3$)

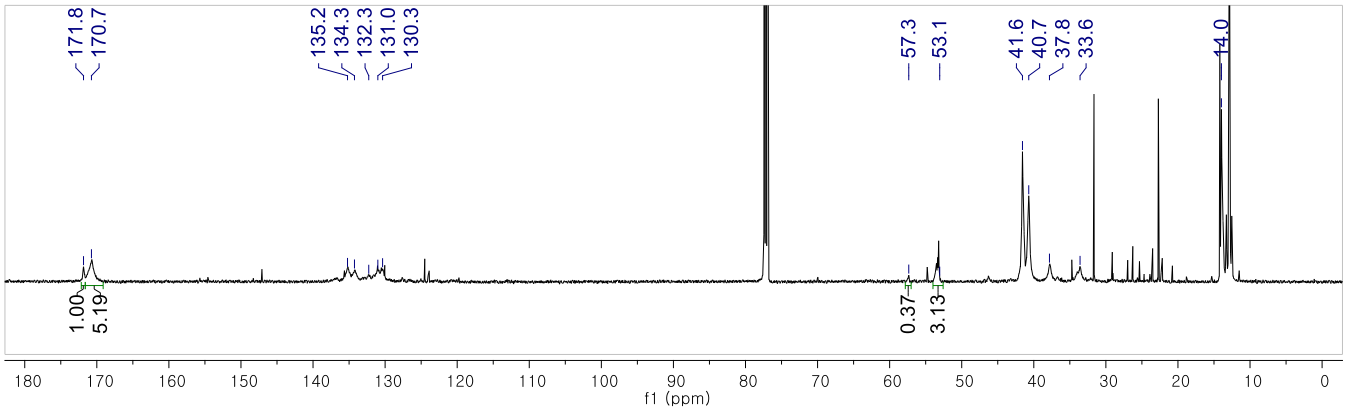

<**P9**-*b*-**P3** from Scheme 3>

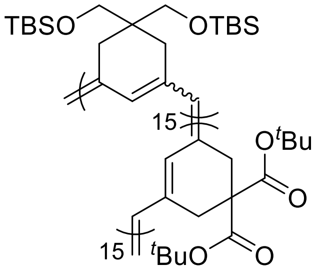

^{1}H NMR (500 MHz, $CDCl_3$)

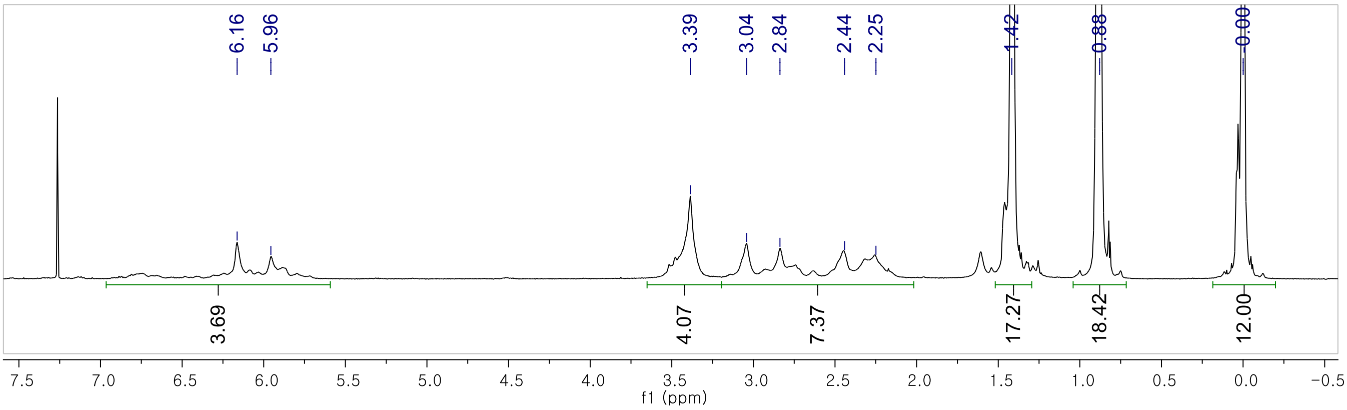

^{13}C NMR (150 MHz, $CDCl_3$)

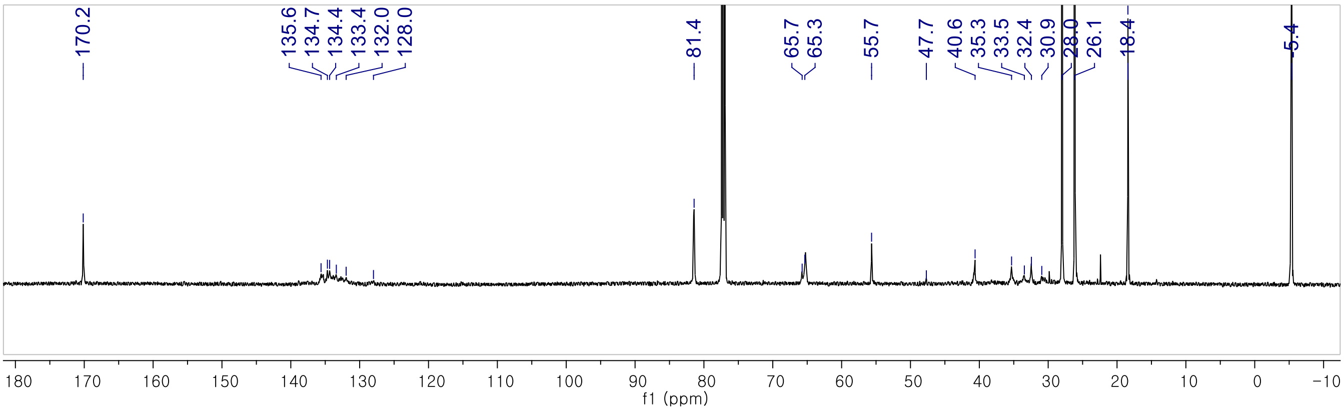

<**P3**-*b*-**P7** from Scheme 3>

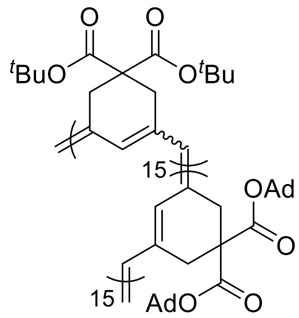

^{1}H NMR (500 MHz, $CDCl_3$)

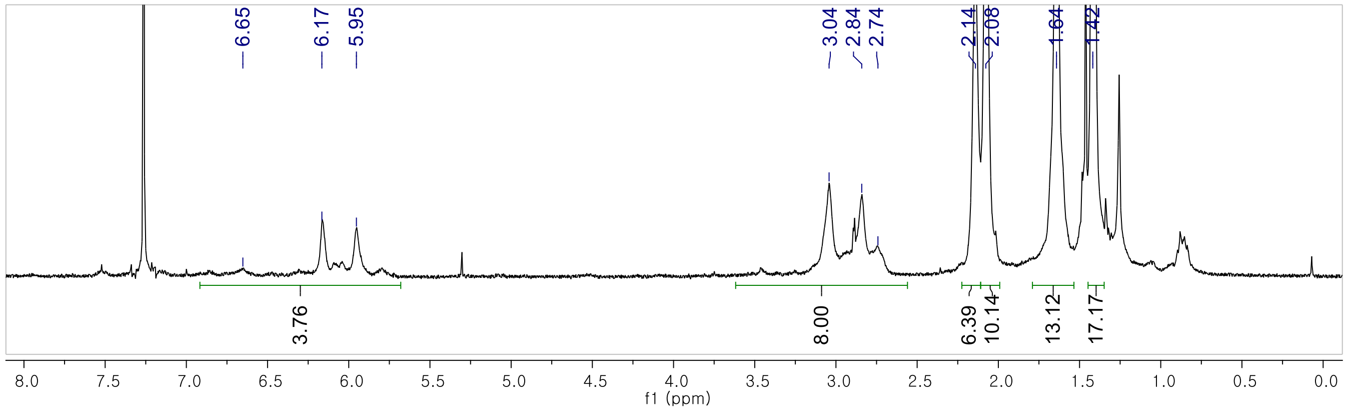

^{13}C NMR (150 MHz, $CDCl_3$)

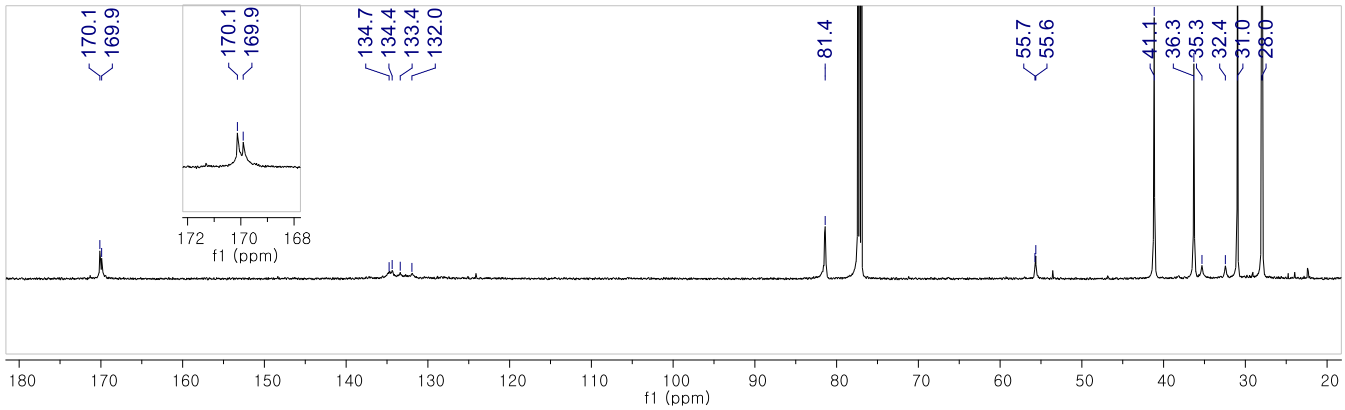

<**P3**-*b*-**P7**-*b*-**P11** from Scheme 3>

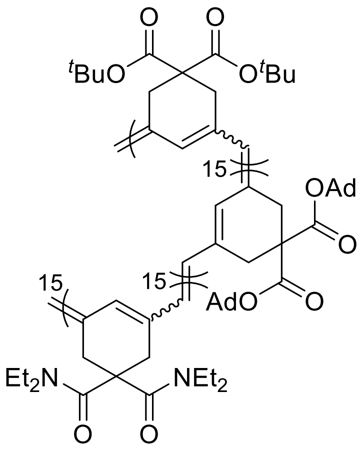

^{1}H NMR (500 MHz, $CDCl_3$)

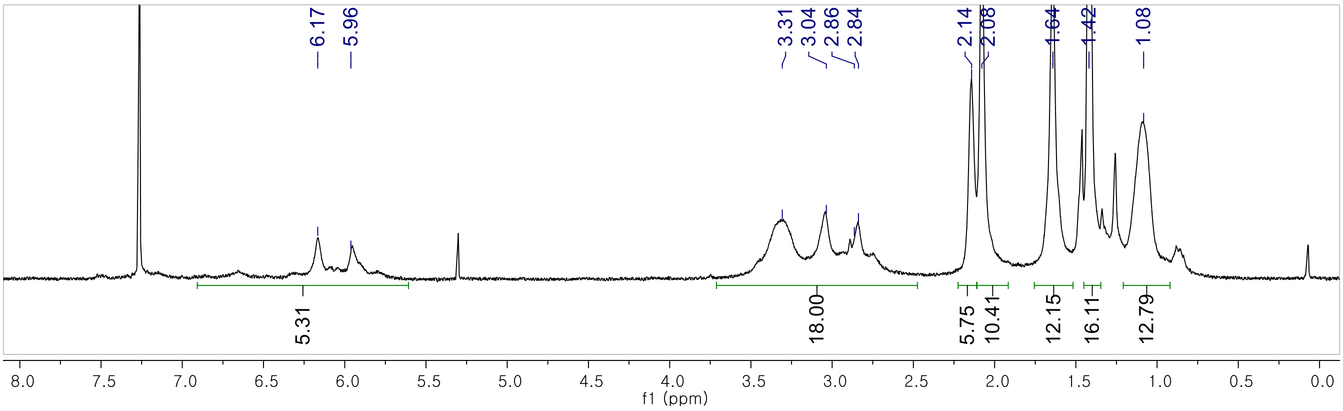

^{13}C NMR (150 MHz, $CDCl_3$)

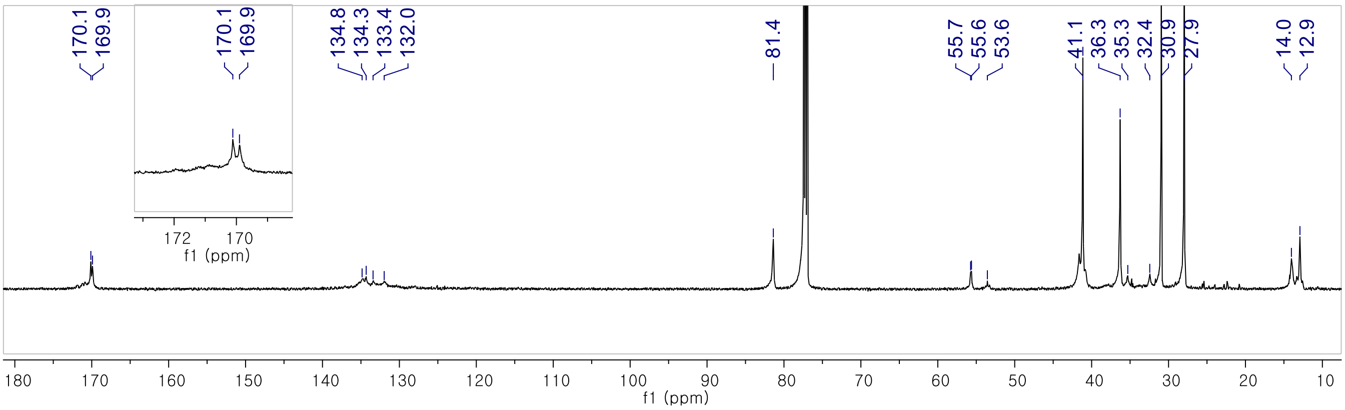

9. ^{1}H and ^{13}C NMR spectra of the monomers

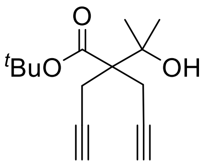

M2-1

^{1}H NMR (500 MHz, $CDCl_3$)

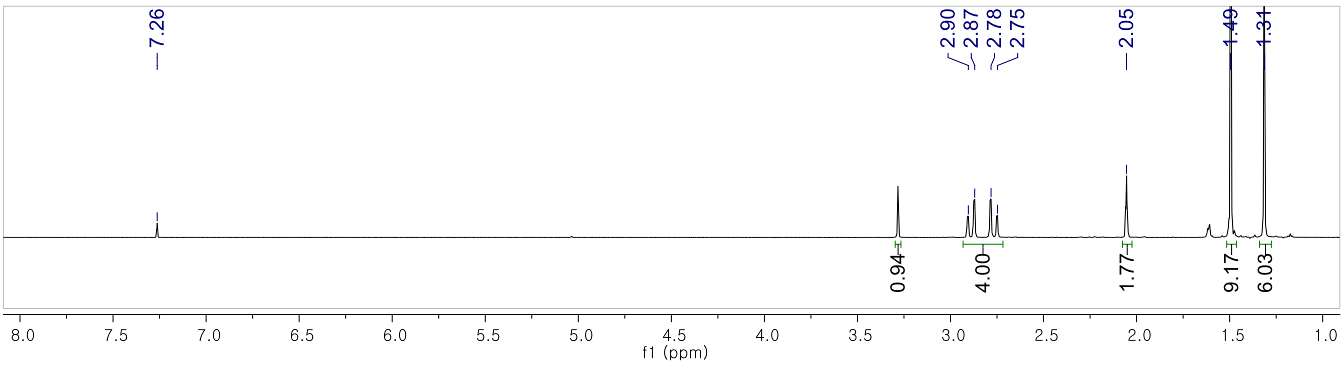

^{13}C NMR (125 MHz, $CDCl_3$)

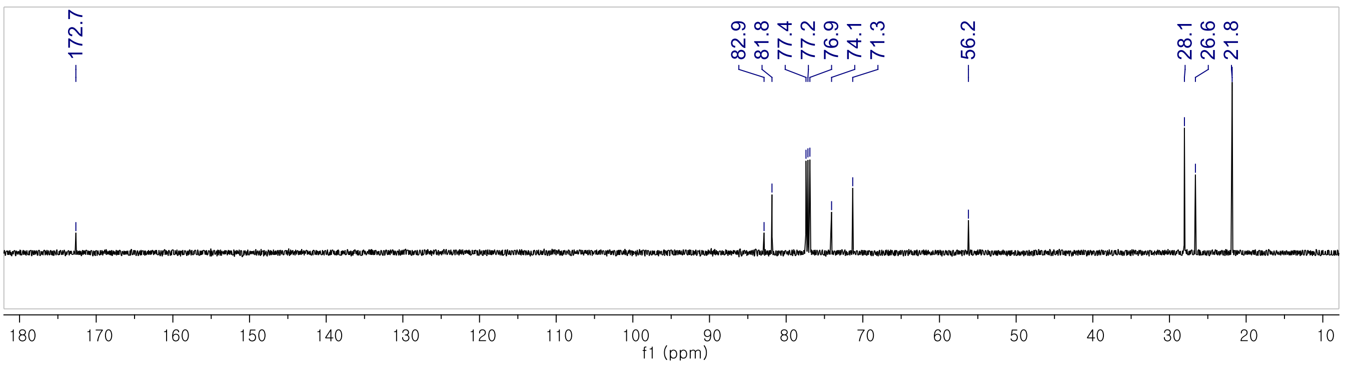

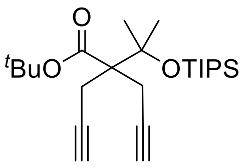

M2

^{1}H NMR (400 MHz, $CDCl_3$)

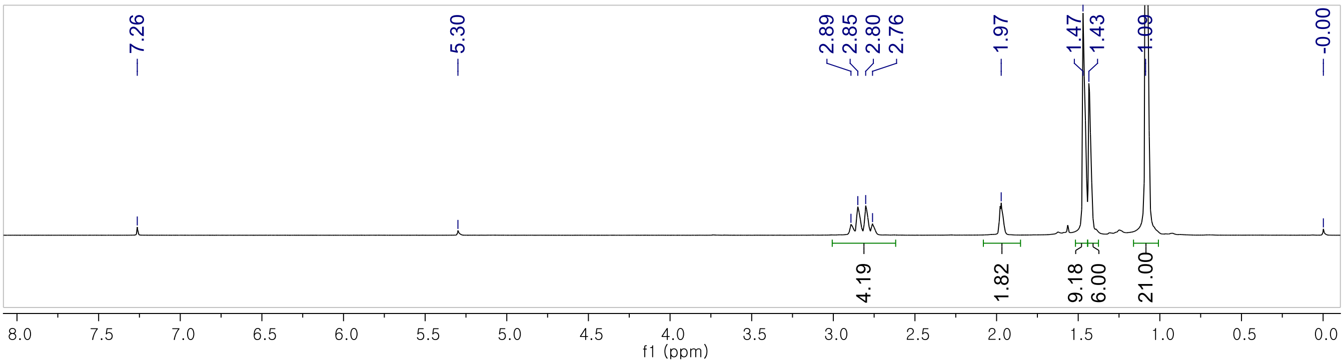

^{13}C NMR (150 MHz, $CDCl_3$)

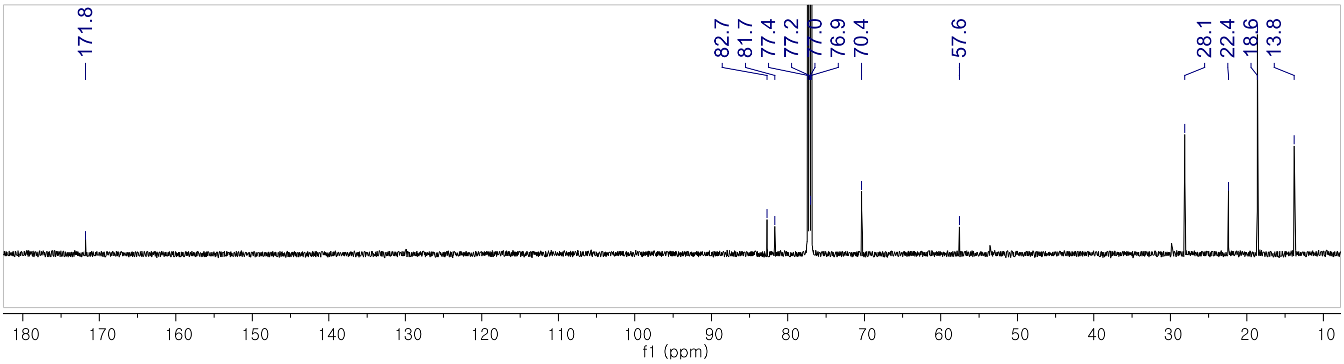

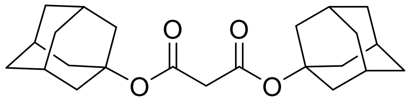

M7-1

^{1}H NMR (500 MHz, $CDCl_3$)

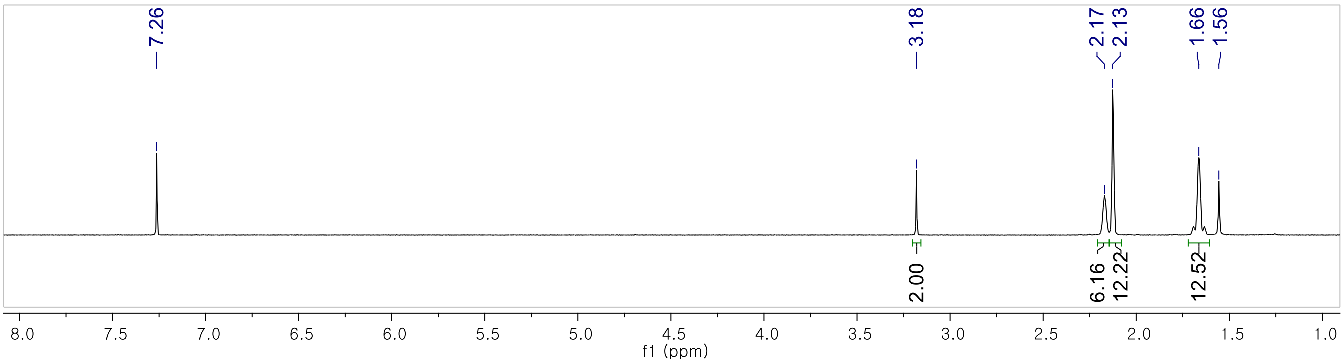

^{13}C NMR (150 MHz, $CDCl_3$)

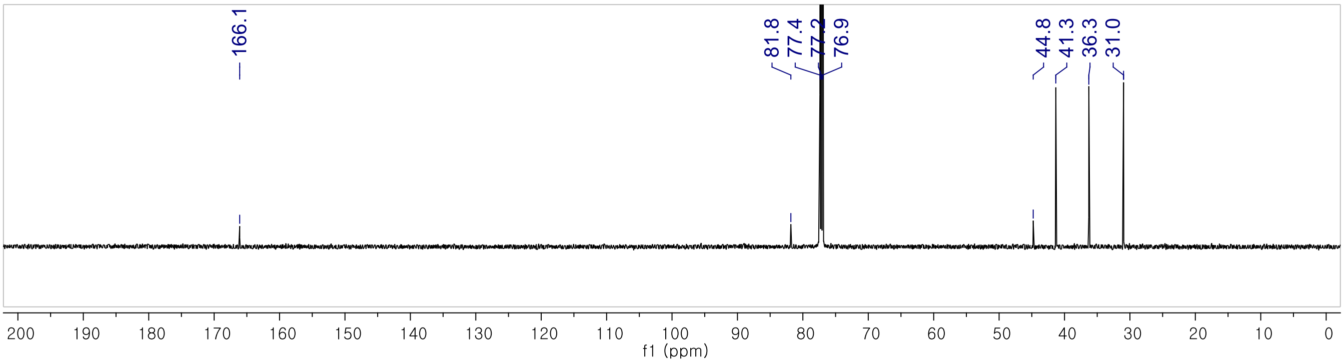

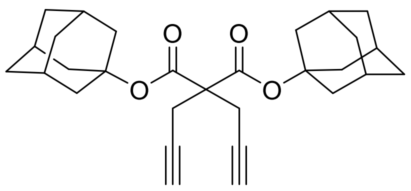

M7

^{1}H NMR (500 MHz, $CDCl_3$)

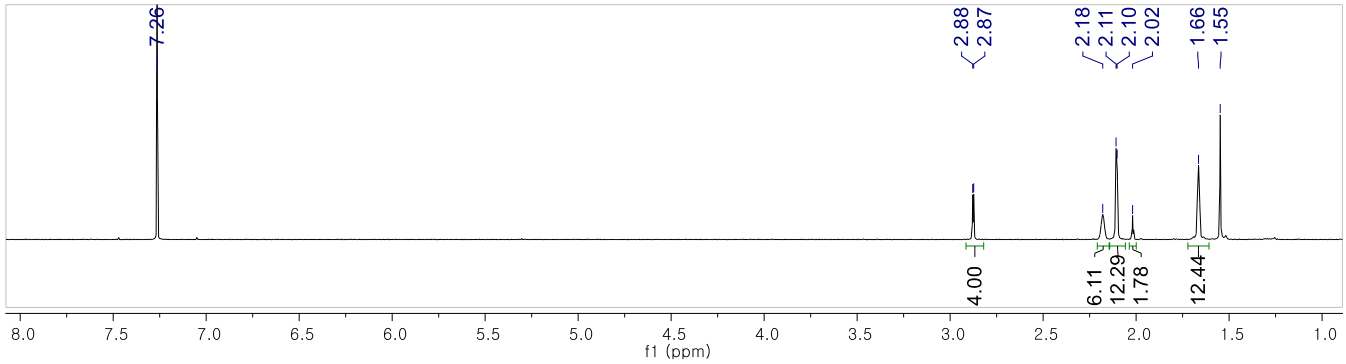

^{13}C NMR (150 MHz, $CDCl_3$)

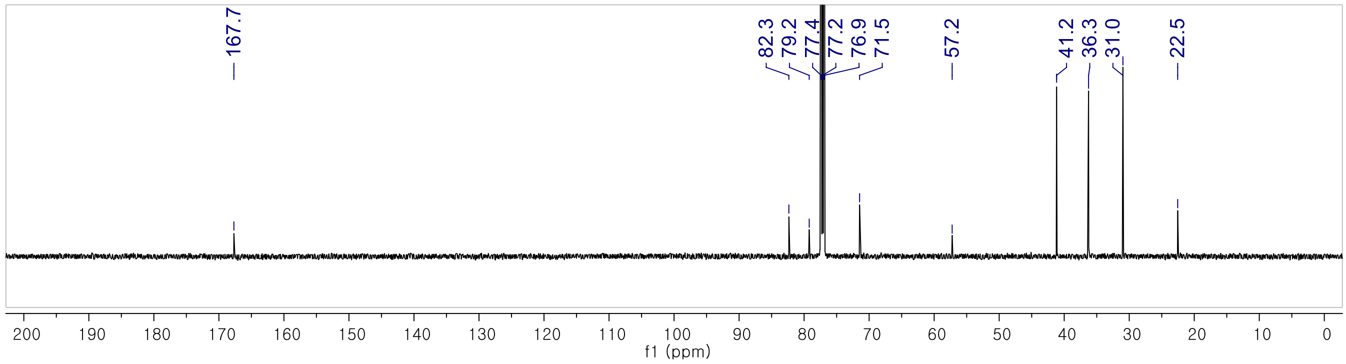

10. MALDI-TOF Spectrum of P2

The molar masses of polymer were measured by Bruker UltrafleXtreme TOF/TOF using dithranol in THF as a matrix. Matrix Assisted Laser Desorption/Ionization Time-of-Flight Mass Spectrometry (MALDI-TOF) spectrum supports that isopropoxy-styrene from **Ru1** remains at one end of the polymer chain, and terminal olefin at the other end of the polymer chain (end-capping was conducted with ethyl vinyl ether).

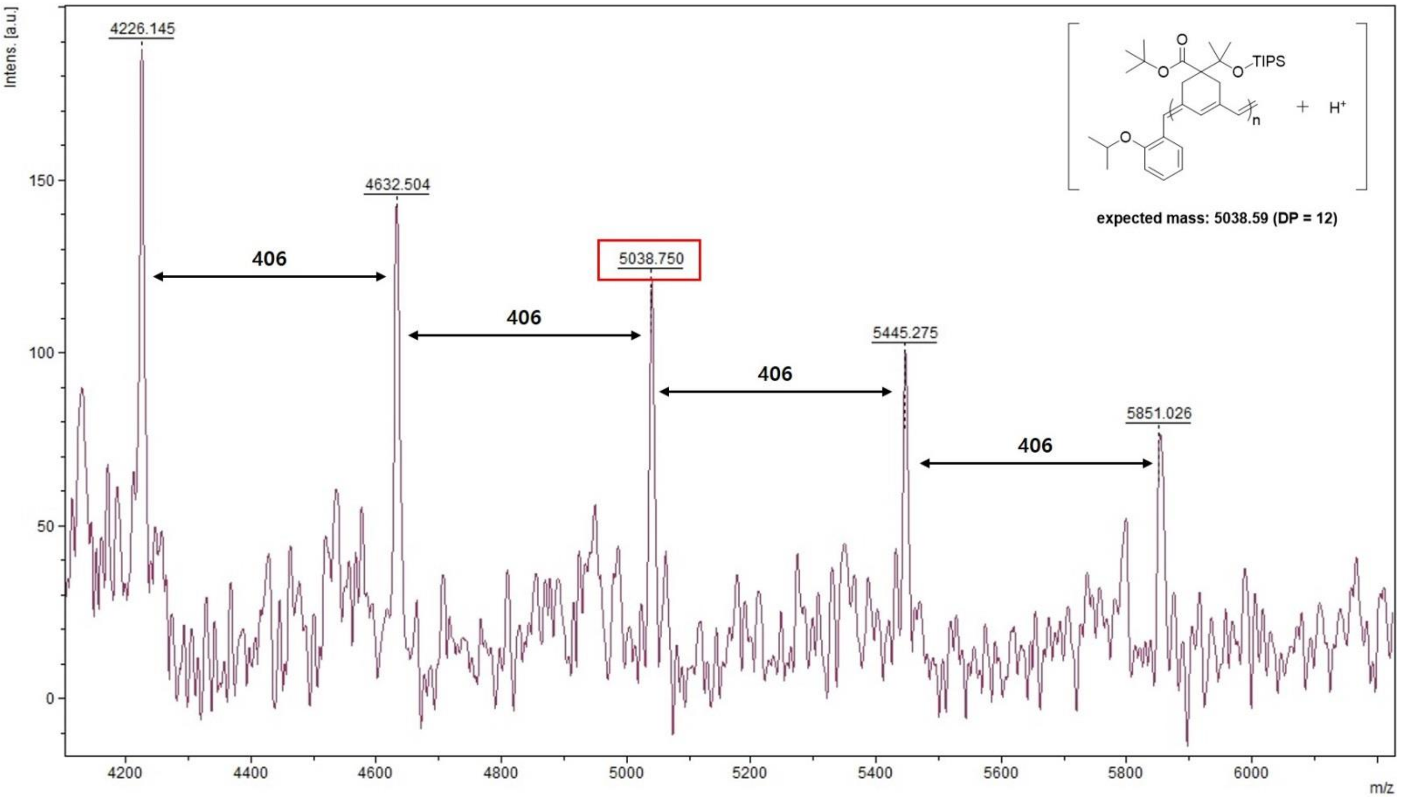

11. References

1. Koh, M. J.; Khan, R. K.; Torker, S.; Yu, M.; Mikus, M. S.; Hoveyda, A. H., High-Value Alcohols and Higher-Oxidation-State Compounds by Catalytic *Z*-Selective Cross-Metathesis. *Nature* **2015,** *517*, 181-186.
2. Ahmed, T. S.; Grubbs, R. H., A Highly Efficient Synthesis of *Z*-Macrocycles Using Stereoretentive, Ruthenium-Based Metathesis Catalysts. *Angew. Chem., Int. Ed.* **2017**, *56*, 11213-11216; Johns, A. M.; Ahmed, T. S.; Jackson, B. W.; Grubbs, R. H.; Pederson, R. L. High *Trans* Kinetic Selectivity in Ruthenium-Based Olefin Cross-Metathesis through Stereoretention. *Org. Lett.* **2016**, *18*, 772-775.
3. Jung, K.; Kim, K.; Sung, J.-C.; Ahmed, T. S.; Hong, S. H.; Grubbs, R. H.; Choi, T.-L., Toward Perfect Regiocontrol for *β*-Selective Cyclopolymerization Using a Ru-Based Olefin Metathesis Catalyst. *Macromolecules* **2018**, *51*, 4564-4571.
